# Wo interacts with SlTCP25 to regulate type I trichome branching in tomato

**DOI:** 10.1093/hr/uhaf032

**Published:** 2025-01-05

**Authors:** Junqiang Wang, Shoujuan Yuan, Yihao Zhao, Xin Shu, Zhiling Liu, Taotao Wang, Zhibiao Ye, Changxian Yang

**Affiliations:** National Key Laboratory for Germplasm Innovation and Utilization of Horticultural Crops, Huazhong Agriculture University, No.1 Shizishan Street, Hongshan District, Wuhan 430070, China; Peking University Institute of Advanced Agricultural Sciences, Shandong Laboratory of Advanced Agricultural Sciences in Weifang, No.699 Binhu Road, Xiashan Eco-Economic Development Zone, Weifang, Shandong 261325, China; National Key Laboratory for Germplasm Innovation and Utilization of Horticultural Crops, Huazhong Agriculture University, No.1 Shizishan Street, Hongshan District, Wuhan 430070, China; Peking University Institute of Advanced Agricultural Sciences, Shandong Laboratory of Advanced Agricultural Sciences in Weifang, No.699 Binhu Road, Xiashan Eco-Economic Development Zone, Weifang, Shandong 261325, China; National Key Laboratory for Germplasm Innovation and Utilization of Horticultural Crops, Huazhong Agriculture University, No.1 Shizishan Street, Hongshan District, Wuhan 430070, China; Peking University Institute of Advanced Agricultural Sciences, Shandong Laboratory of Advanced Agricultural Sciences in Weifang, No.699 Binhu Road, Xiashan Eco-Economic Development Zone, Weifang, Shandong 261325, China; National Key Laboratory for Germplasm Innovation and Utilization of Horticultural Crops, Huazhong Agriculture University, No.1 Shizishan Street, Hongshan District, Wuhan 430070, China; Peking University Institute of Advanced Agricultural Sciences, Shandong Laboratory of Advanced Agricultural Sciences in Weifang, No.699 Binhu Road, Xiashan Eco-Economic Development Zone, Weifang, Shandong 261325, China; National Key Laboratory for Germplasm Innovation and Utilization of Horticultural Crops, Huazhong Agriculture University, No.1 Shizishan Street, Hongshan District, Wuhan 430070, China; Peking University Institute of Advanced Agricultural Sciences, Shandong Laboratory of Advanced Agricultural Sciences in Weifang, No.699 Binhu Road, Xiashan Eco-Economic Development Zone, Weifang, Shandong 261325, China; National Key Laboratory for Germplasm Innovation and Utilization of Horticultural Crops, Huazhong Agriculture University, No.1 Shizishan Street, Hongshan District, Wuhan 430070, China; National Key Laboratory for Germplasm Innovation and Utilization of Horticultural Crops, Huazhong Agriculture University, No.1 Shizishan Street, Hongshan District, Wuhan 430070, China; National Key Laboratory for Germplasm Innovation and Utilization of Horticultural Crops, Huazhong Agriculture University, No.1 Shizishan Street, Hongshan District, Wuhan 430070, China; Peking University Institute of Advanced Agricultural Sciences, Shandong Laboratory of Advanced Agricultural Sciences in Weifang, No.699 Binhu Road, Xiashan Eco-Economic Development Zone, Weifang, Shandong 261325, China

## Abstract

Plant trichomes serve as a protective barrier against various stresses. Although the molecular mechanisms governing the initiation of trichomes have been extensively studied, the regulatory pathways underlying the trichome branching in tomato remain elusive. Here, we found that *Woolly* (*Wo*) mutant and its overexpression transgenic plants displayed branched type I trichomes*.* The expression level of *SlTCP25*, a transcription factor of type TB1 of the TCP subfamily, was obviously decreased in *Wo* mutant and *Wo* overexpressing lines. Knockout of *SlTCP25* resulted in the formation of type I trichome branches on the hypocotyls. Genetic evidence showed that *SlTCP25* is epistatic to *Wo* in the branched trichome formation. Biochemical data further indicated that Wo can directly bind to the L1-box *cis-*element in the *SlTCP25* promoter and repress its transcription. We further determined that SlTCP25 interacts with Wo to weaken Wo-regulated the expression of *SlCycB2*, a trichome branching inhibitor*.* In addition, the number of trichome branches was significantly increased in *Sltcp25Slcycb2* double mutant, suggesting that SlTCP25 and SlCycB2 coordinately repress trichome branching in wild type. In conclusion, we elucidate a molecular network governing the morphogenesis of multicellular trichomes in tomato.

## Introduction

Trichomes, also known as epidermal hairs, serve as the outermost protective tissue in terrestrial plants. They play a crucial role in protecting plants against various adverse factors, including extreme temperatures, ultraviolet radiation and herbivorous pests [1–3]. Trichomes can be classified as unicellular or multicellular, glandular or non-glandular, and branched or non-branched [4, 5]. There are unicellular trichomes with three to four branches in Arabidopsis, making it an excellent system for studying cell fate determination and polarity [6]. Mature trichomes undergo three developmental stages in Arabidopsis: cell fate determination and initiation, branch formation, and trichome elongation and maturation [7, 8].

Plant-specific TCP transcription factors play important roles in plant growth and development [9, 10]. According to the difference in TCP domains, the TCP family members can be divided into two categories, Class I and Class II, and the later one was further divided into CIN and CYC/TB1 subfamilies [9]. Recent studies showed that *TCP4* inhibits the formation of trichome branches by activating the expression of *GLABROUS INFLORESCENCE STEMS* (*GIS*), which functions as a negative regulator of trichome branching in Arabidopsis [11, 12]. In addition, TCP4 interacts with GL3 to inhibit the activation function of GL3-GL1-TTG1 complex, which result in the obstruction of the initiation process of leaf trichomes [13]. Inhibition of *TCP14* or *TCP15* expression results in an increase in the number of trichome branches, similar to the phenotype of the *tcp14tcp15* double mutant [14–16]. In cotton, *GbTCP* is specially expressed in long cotton fibers, and the inhibition of *GbTCP* expression results in shorter and lower quality cotton fibers than those of wild-type, while its overexpression in Arabidopsis promotes root hair initiation and trichome branching [17]. Overexpression of *GbTCP5* in Arabidopsis results in a significant increase of root hairs and stem trichomes. Biochemical experiments showed that GbTCP5 regulates the expression of *GL3*, *EGL3*, *CPC*, and other trichome regulators [18]. Trichome branching is also regulated by other transcription factors in Arabidopsis, such as HD-ZIP family member *GLABR2* (*GL2*) and bHLH family member *GLABR3* (*GL3*). Trichomes exhibit unbranched or reduced branches in the *gl2* or *gl3* mutant [19–21].

Tomato trichomes are multicellular and non-branched, which can be classified into seven types [22]. Among them, types I–V are digitate trichomes, while type VI and VII are peltate trichomes [23]. *Woolly* (*Wo*), an HD-Zip IV transcription factor, regulates the early development of type I trichomes in a dose-dependent manner [23, 24]. Overexpression of *Wo* results in a significant increase in the number of trichomes on stems and leaves. On the contrary, overexpression of *SlCycB2* or *SlCycB3* results in the loss of trichomes, especially type I trichomes [24, 25]. A recent study revealed that multicellular trichome repressor 1 (*MTR1*), previously known as *SlCycB2* that contains an EAR and a RING-like domain, negatively regulates the expression of Wo at protein level [23]. *Hair* (*H*), encoding a C2H2 transcription factor, positively regulates type I trichome formation. Biochemical experiments confirmed the physical interaction between Wo and H [26]. Moreover, Wo also interacts with an HD-ZIP transcription factor Lanata (Ln) to enhance the expression of *SlCycB2* and *SlCycB3*, resulting in increased trichome density [27].

**Figure 1:**
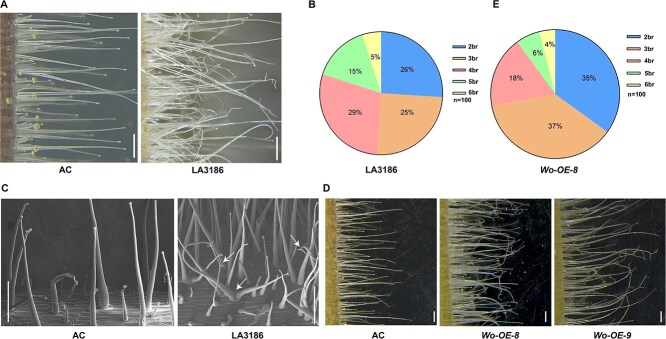
Phenotypic characterization of AC, *Wo* mutant, and *Wo* overexpression plants. (A) Hypocotyl trichomes of AC and LA3186 plants. Bar, 500 μm. (B) The branch percentage of the type I trichomes on hypocotyl of LA3186. br, branch number per trichome (n = 100). (C) Images of the hypocotyl trichomes of AC and LA3186. The images were obtained by cryo-scanning electron microscopy. The white arrows indicate the branch points. Bar, 500 μm. (D) Hypocotyl trichomes of AC and *Wo* overexpression lines. Bar, 500 μm. (E) The branch percentage of type I trichomes on the hypocotyl of *Wo* overexpression lines. br, branch number per trichome (n = 100).

Several regulators are involved in the morphogenesis of trichomes in tomato. For example, overexpression of *SlMX1*, a MYB transcription factor, promotes type III-like trichome branching [28, 29]. SlMixta-like is specifically expressed in trichomes, and knockout of *SlMixta-like* causes the malformation of the majority of trichomes [30, 31]. *Hl* encoding a subunit of the WAVE regulatory complex, the mutation of which caused distorted trichome phenotype [32]. Suppression of *HD-ZIPIV8* expression leads to the curved growth of trichomes, similar to the phenotype of *Hairless 2* (*Hl-2*) mutant [33]. Single nucleotide mutation in *hairless 3* (*hl-3*) leads to the swelling and curve trichomes [34].

In this study, we determined that *Wo* is a key regulator of type I trichome branching. *SlTCP22* and its close homolog *SlTCP25* also regulate the formation of trichome branches. The interaction among Wo, SlTCP25, and SlTCP22 suggests that they may collectively control trichome branching. In addition, SlTCP25 interacts with Wo to affect the expression level of *SlCycB2*, which is a downstream target of Wo. These findings reveal an important role of Wo-SlTCP25 module in trichome branching and broaden our knowledge on the molecular mechanism underlying trichome cell morphogenesis in tomato.

## Results

### *Wo* modulates type I trichome branching

Trichomes were classified into seven types (I–VII) in tomato [22]. Previous studies indicated that *Wo* regulates the initiation of type I trichomes [24]. Whether *Wo* regulates the morphogenesis of trichomes has not been characterized. Here, we found that the majority of type I trichomes on the hypocotyls of *Wo* mutant LA3186 displayed branches, whereas no branched trichomes were observed on those of AC (Fig. 1A). Quantification data showed that type I trichomes on the hypocotyls of LA3186 had about two to six branches (Fig. 1B). However, no obvious branched trichomes were observed on the leaves and stems of LA3186 and AC (Fig. S1). Cryo-SEM observation revealed that branch points are initiated on the upper, middle and lower positions of type I trichomes in LA3186, in which overall orientation of the trichomes remains upright (Fig. 1C). Moreover, we observed the phenotype of type I trichomes of LA3560 (another gain-of-function mutant of *Wo*) [35]. Compared to AC, LA3560 trichomes exhibited more branches on hypocotyls (Fig. S2A). There are approximately two to nine branches on the type I trichomes of LA3560 (Fig. S2B). These data suggest that Wo may play a critical role in trichome branch formation.

To further confirm the role of *Wo* in modulating trichome branch formation, we generated *Wo* overexpression lines (*Wo-OE*) driven by CaMV 35S promoter in AC. Similar to LA3186, the type I trichomes exhibited two to six branches in *Wo-OE* lines (Fig. 1D and E). Taken together, these results suggest that *Wo* positively regulates the branch formation of trichomes in tomato.

**Figure 2:**
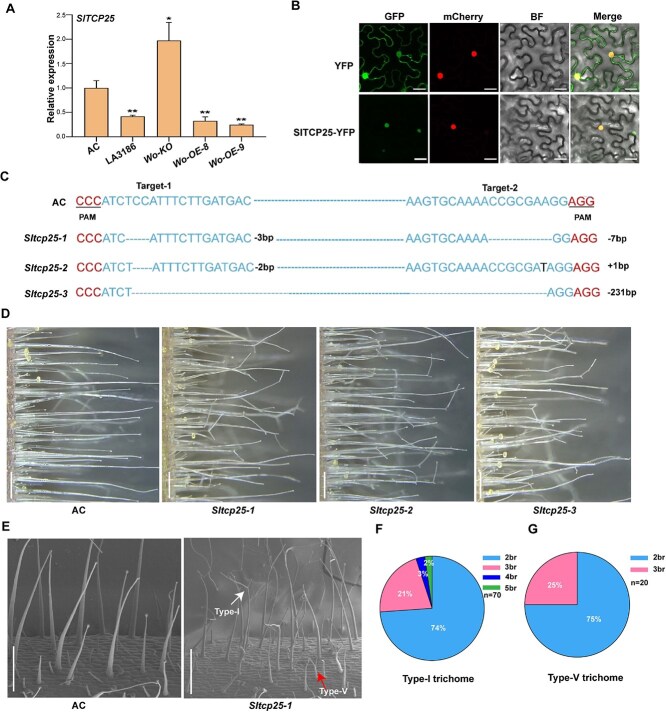
Functional validation of *SlTCP25*. (A) Expression levels of *SlTCP25* in AC, LA3186, *Wo-KO*, and *Wo-OE* plants. RNA samples were taken from hypocotyl trichomes. Data are means ± SD (n = 3). The *p*-values were determined using the Student’s *t-test* (^*^, *P* < .05; ^**^, *P* < .01). (B) Subcellular localization of *SlTCP25* in *N. benthamiana* leaf epidermal cells. YFP indicates empty vector with a yellow fluorescent protein tag. GFP, green fluorescent protein; mCherry, NLS-RFP fusion protein with red fluorescent; BF, bright field. The images were captured using a laser confocal microscope. Bar, 25 μm. (C) Gene editing results of three *Sltcp25* lines. The letters with underline represent the PAM sites. The inserted (+) and deleted (−) bases are presented on the right side of each target site. (D) Trichomes of AC and the *Sltcp25* lines. The samples were collected from three-week-old hypocotyls of AC and the *Sltcp25* lines. Bar, 500 μm. (E) Images of the hypocotyl trichomes of AC and the *Sltcp25* plants from cryo-SEM. Bar, 500 μm. (F) and (G) The branch percentage of the type I trichomes (F) and type V trichomes (G) on the hypocotyl of the *Sltcp25* lines. br, branch number per trichome (n = 70 in F, n = 20 in G).

**Figure 3:**
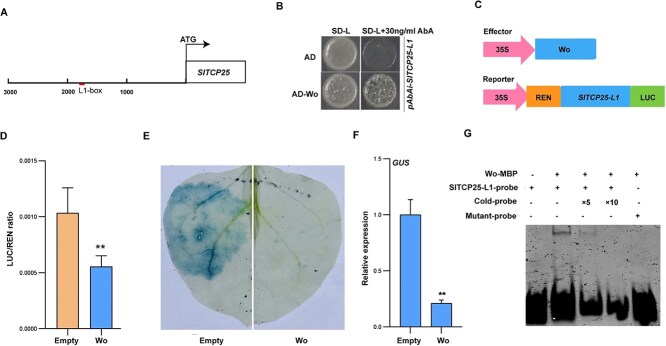
Wo inhibits the expression of *SlTCP25*. (A) Schematic diagram of the 3 kb promoter sequence of *SlTCP25*. The underline indicates the L1-box *cis-*element location in the promoter of *SlTCP25*. (B) Wo binds to the *SlTCP25* promoter, as shown by Y1H assay. Co-expression of empty AD with the *SlTCP25* promoter was used as a negative control. Yeast cells were grown and selected on SD-Leu medium containing 30 ng/ml aureobasidin A (AbA). (C) Schematic diagram of Wo effector construct and *LUC* gene reporter construct. (D) Dual luciferase assay showing the effect of Wo on the activity of the *SlTCP25* promoter in *N. benthamiana* leaves, as confirmed by co-expression of empty pGreen II 62-SK (Empty) or 62SK-Wo (Wo) and *proSlTCP25*: *LUC*. *LUC,* firefly luciferase activity; *REN*, Renilla reniformis luciferase activity. Data are means ± SD (n = 3). Asterisks indicate significant differences according to the Student’s *t-test* analysis (^**^, *P* < .01). (E) and (F) Analysis of β-glucuronidase (*GUS*) activity after co-expression Wo effector protein and the *SlTCP25* promoter. Transient expression of *GUS* gene driven by the *SlTCP25* promoter in *N. benthamiana* leaves (E) was utilized for quantitative analysis of *GUS* expression (F). Data are means ± SD (n = 3). The empty vector driven by 35S was used as a negative control. The *p*-values were determined using the Student’s *t-test* (^**^, *P* < .01). (G) EMSA assay showed that Wo binds to L1-box element in the promoter of *SlTCP25*. The black bands below represent the probes, and the bands above indicate the mixture of target protein and the probes. The SlTCP25-L1-probe (CATAAAAGGTATGGTAAATGCTATCAGTATATAT) and mutated probe (CATAAAAGGTATGGTTTTTTTTATCAGTATATAT) were used for the assay. Probes are labeled with 5’ FAM.

### Knockout of *SlTCP25* gives rise to branched trichomes

In Arabidopsis, several members of TCP family are involved in the regulation of trichome branch formation, such as *TCP4*, *TCP14*, and *TCP15* [12, 36]. However, *SlTCP25* showed highest sequence similarity with Arabidopsis *TCP1* (Fig. S3B), which mainly regulate plant growth and development [37]. qRT-PCR showed that the expression level of *SlTCP25* was significantly decreased in LA3186 and *Wo-OE* plants but increased in *Wo-KO* (*wo*) plants compared to AC (Fig. 2A) Sequence alignment showed that the amino acid sequence encoded by *SlTCP25* contains a conserved bHLH domain (Fig. S3A). Subcellular localization analysis revealed that the SlTCP25-YFP fusion protein was localized in the nucleus (Fig. 2B). Tissue expression analysis showed that *SlTCP25* is highly expressed in trichomes, suggesting that *SlTCP25* may be involved in the development of trichomes (Fig. S4).

To investigate the function of *SlTCP25* in regulating the formation of trichome branches, we generated *SlTCP25* knockout lines in AC background using the CRISPR/Cas9 system. Three homozygous mutant lines (*Sltcp25–1*, *Sltcp25–2,* and *Sltcp25–3*) were obtained for further characterization (Fig. 2C). Compared to AC plants, all *Sltcp25* lines exhibited branched trichomes on the hypocotyls (Fig. 2D and E). Quantification analyses showed that there are two to five branches of the type I trichomes in the *Sltcp25* lines (Fig. 2F), similar to that in LA3186 and *Wo-OE* lines. In addition, we also found that a few type V trichomes have branches (Fig. 2E and G). In the *Sltcp25* lines, the branch points of the trichome are initiated mainly on the upper-middle region, the 3rd and 6th cells of type I trichomes (Fig. S5A). Trichomes had very few branches on the stems and leaves of *Sltcp25* lines (Fig. S5B). These data suggest that *SlTCP25* inhibits the formation of trichome branches in tomato.

### Wo affects trichome branching by repressing *SlTCP25* transcription

The expression of *SlTCP25* was repressed in LA3186 and *Wo-OE* plants (Fig. 2A), suggesting that *SlTCP25* may be a target of Wo. We thus analyzed the 3 kb promoter region of *SlTCP25* and found one putative L1-box element (TAAATGCT), a major binding site of HD-ZIP IV transcription factors (Fig. 3A) [38]. To examine whether Wo binds to *SlTCP25* promoter, we conducted a yeast one-hybrid (Y1H) assay. The results demonstrated that the yeast cells expressing pGADT7-Wo and pAbAi-*SlTCP25* could grow on SD/−Leu deficient medium containing 30 ng/ml AbA, while negative control failed to grow on the same media, suggesting that Wo can bind to the promoter of *SlTCP25* (Fig. 3B). Moreover, electrophoretic mobility shift assay (EMSA) assay indicated that the increasing amounts of cold probe evidently decreased the ability of Wo binding to the FAM-labeled probes, suggesting that the Wo-MBP fusion protein could bind to this L1-box element (Fig. 3G). Subsequently, a dual-luciferase assay was performed. When *Wo* was co-expressed with *LUC* gene driven by *SlTCP25* promoter in *Nicotiana benthamiana* leaves, the ratio of LUC/REN was obviously reduced compared to empty control, suggesting that Wo negatively regulates the expression of *SlTCP25* in planta (Fig. 3, C and D). The *GUS* activity analysis further confirmed that *SlTCP25* expression was strongly repressed by Wo, consistent with the above result (Fig. 3, E and F). These results demonstrate that Wo directly binds to L1-box element of *SlTCP25* promoter to repress its expression.

**Figure 4:**
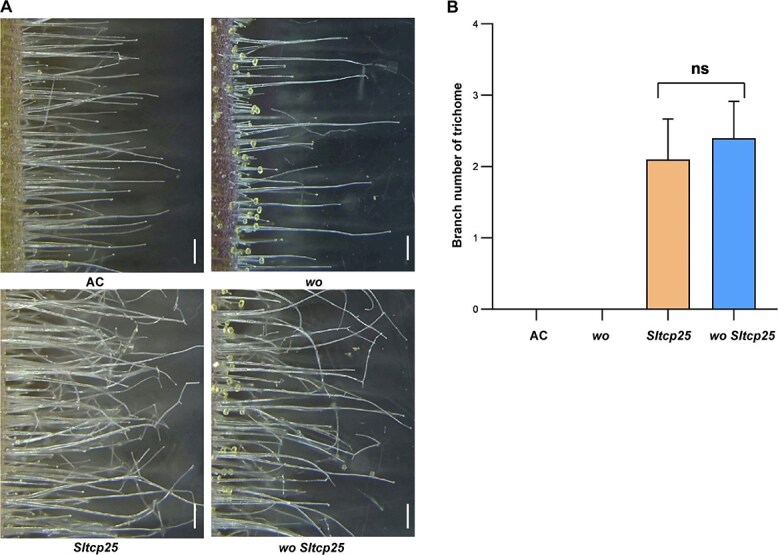
Genetic interaction of Wo and SlTCP25 during trichome branch formation. (A) Trichome phenotype of AC, *wo* mutant, *Sltcp25* mutant*,* and the *wo Sltcp25* double mutant. The representative images are presented from three-week-old hypocotyls. Bar, 500 μm. (B) The average branch number of the type I trichomes on hypocotyl of the genotypes in (A). Data are means ± SD (n = 10). Statistically significant differences were determined using Student’s *t-test*.

To investigate the genetic relationship between *Wo* and *SlTCP25*, a cross between *Wo* knockout mutant lines (*wo*) and *Sltcp25* lines was conducted. The *wo Sltcp25* double mutants were obtained by screening F_2_ segregating individuals. The number of trichome branches of *wo Sltcp25* double mutants was similar to that of *Sltcp25* lines, but markedly different from those of AC and *wo* plants (Fig. 4, A and B), suggesting that *Wo* is dependent on *SlTCP25* in modulating trichome branch formation. Overall, genetic and biochemical experiments suggest that *Wo* acts upstream of *SlTCP25* and represses its expression.

### Wo physically interacts with SlTCP25

**Figure 5:**
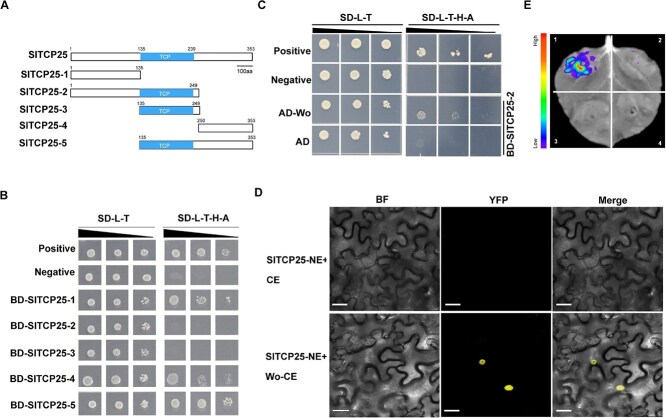
SlTCP25 interacts with Wo. (A) Schematic diagram of the truncated SlTCP25 amino acid sequence. SlTCP25–1, N-terminal; SlTCP25–2, N-terminal containing TCP domain; SlTCP25–3, single TCP domain; SlTCP25–4, C-terminal; SlTCP25–5, C-terminal containing TCP domain. (B) Self-activation assay of SlTCP25 in yeast. The truncated SlTCP25 fragments were cloned into the pGBKT7 vector, respectively, and the resulting recombinant plasmids were separately transformed with empty pGADT7 into yeast strain AH109. The yeast cells were cultured on SD-Leu-Trp (SD-L-T) and selected on SD-Leu-Trp-His-Ade (SD-L-T-H-A) deficient media. Positive, BD-p53 + AD-T; Negative, BD-lam + AD-T. (C) Y2H assay showing the interaction between Wo and SlTCP25. Positive, BD-p53 + AD-T; Negative, BD-lam + AD-T and BD-SlTCP25–2 + AD. Three biological replicates were performed in each group. (D) BiFC assay analysis of the interaction between Wo and SlTCP25. The fluorescence signal indicates that Wo interacts with SlTCP25. SlTCP25-NE and CE combinations were used as a negative control. Bar, 25 μm. (E) LCI assay showing the protein interaction between Wo and SlTCP25. The combinations represented by different numbers are as follows: (1) Wo-nLUC+SlTCP25-cLUC; (2) Wo-nLUC+cLUC; (3) nLUC+SlTCP25-cLUC; (4) nLUC+cLUC. Compared to the other control groups, the fluorescence signals indicate the interaction between Wo and SlTCP25.

To further understand the relationship between Wo and SlTCP25 at molecular level, we tested the possible physical interaction between Wo and SlTCP25 through yeast two-hybrid (Y2H) assay. At first, we conducted a yeast self-activation test through a truncation experiment (Fig. 5A). The yeast cells containing the N-terminal region with TCP domain (SlTCP25–2) and single TCP domain (SlTCP25–3) failed to grow on SD/−L-T-H-A medium, indicating that these fragments had no self-activating activity (Fig. 5B). The Y2H assay showed that Wo was able to interact with SlTCP25–2 in yeast (Fig. 5C). In a bimolecular fluorescence complementation (BiFC) assay, the yellow fluorescence signal was detected in the nucleus of *N. benthamiana* leaves when Wo-CE was co-expressed with SlTCP25-NE in *N. benthamiana*, confirming the interaction between Wo and SlTCP25 in planta (Fig. 5D). We further confirmed the interaction between them using a luciferase complementation imaging (LCI) assay. A strong fluorescent signal was detected when *Wo-nLUC* and *SlTCP25-cLUC* were co-expressed in *N. benthamiana* leaves, while no fluorescence was observed in negative control (Fig. 5E). Taken together, these results indicate that Wo physically interacts with SlTCP25.

### SlTCP22 and SlTCP25 interact with each other and have a functional redundancy role in trichome branch formation

Given the high amino acid similarity between SlTCP25 and SlTCP22 (Fig. S3B), we hypothesized that *SlTCP22* may have a function similar to that of *SlTCP25*. qRT-PCR results showed that the transcript level of *SlTCP22* was significantly reduced in LA3186 compared to AC, especially in the hypocotyl trichomes (Fig. S6A). To gain insight into the role of *SlTCP22* in trichome branch formation, we generated *SlTCP22* knockout mutants (*Sltcp22*) in AC. Compared to AC plants, the trichomes on the hypocotyls of the *Sltcp22* lines produced branches (Fig. S6B), which are similar to a previous study [39]. The majority of trichomes had two to four branches, which were predominantly initiated at the 3rd and 6th cells of type I trichomes in the *Sltcp22* lines (Fig. S6C and D). These results suggest that *SlTCP22* and *SlTCP25* have a similar role in regulating trichome branch formation.

**Figure 6:**
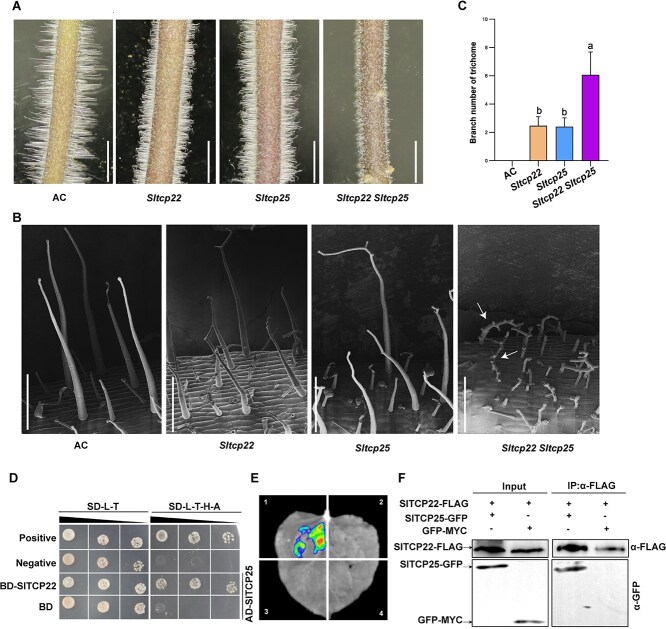
The interaction of SlTCP22 and SlTCP25. (A) and (B) Trichome phenotype of AC, *Sltcp22* mutant, *Sltcp25* mutant, and the *Sltcp22 Sltcp25* double mutant. Bar, 5 mm in A, 500 μm in B. (C) The average branch number of the type I trichomes on hypocotyl of the AC, *Sltcp22*, *Sltcp25*, and the *Sltcp22 Sltcp25* double mutant. Data are means ± SD (n = 20). Statistically significant differences were determined using one-way ANOVA. (D) Y2H assay showing the interaction of SlTCP25 and SlTCP22 in yeast. Positive, BD-p53 + AD-T; Negative, BD-lam + AD-T and empty BD. (E) Analysis of the interaction between SlTCP22 and SlTCP25 in LCI assay. The combinations represented by different numbers are as follows: (1) SlTCP25-nLUC+SlTCP22-cLUC; (2) SlTCP25-nLUC+cLUC; (3) nLUC+SlTCP22-cLUC; (4) nLUC+cLUC. (F) Interaction of SlTCP25 and SlTCP22 in *N. benthamiana* leaf cells, as validated by co-immunoprecipitation assay. The total proteins and immunoprecipitated proteins with anti-FLAG agarose beads were detected by western blotting using anti-GFP antibody and anti-FLAG antibody, respectively. GFP-MYC protein driven by 35S promoter was used as a negative control.

To understand the genetic relationship between *SlTCP22* and *SlTCP25*, we generated a double mutant by crossing *Sltcp22* lines with *Sltcp25* lines. Compared to the *Sltcp22* lines or *Sltcp25* lines, the number of trichome branches in *Sltcp22Sltcp25* double mutants was significantly increased (Fig. 6A–C), suggesting that *SlTCP22* and *SlTCP25* are functionally redundant in the regulation of trichome branching.

Previous studies showed that Wo could interact with SlTCP22 [39]. It is worthwhile to detect the potential interaction between SlTCP22 and SlTCP25. As expected, Y2H assay showed that SlTCP22 directly interacts with SlTCP25, consistent with the results of LCI assay (Fig. 6D and E). The interaction was further verified by co-immunoprecipitation (Co-IP) assay. When SlTCP25-GFP and SlTCP22-FLAG were co-expressed in *N. benthamiana* leaves, both proteins were co-immunoprecipitated together (Fig. 6F). Together, these results indicate that SlTCP22 and SlTCP25 interact with each other.

### *SlTCP25* and *SlCycB2* coordinately inhibit trichome branching

It has been shown that *SlCycB2* negatively regulates the initiation of trichomes [25]. To further investigate the role of *SlCycB2* in trichome branch formation, three homozygous mutant lines (*Slcycb2*–2, *Slcycb2*–7, and *Slcycb2*–10) were obtained using CRISPR/Cas9 system in AC background (Fig. S7A). All *Slcycb2* lines also displayed branched trichomes on the hypocotyls (Fig. S7B and C). The majority of trichomes had two to five branches in the *Slcycb2* lines (Fig. S7D). These results suggest that *SlCycB2* acts an important negative regulator of trichome branch formation.

**Figure 7:**
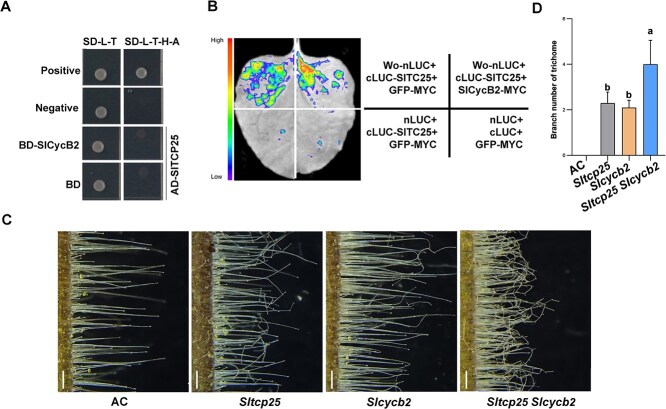
The molecular and genetic relationship of *SlTCP25* and *SlCycB2*. (A) SlTCP25 do not interact with SlCycB2 in yeast, as shown by Y2H assay. Positive, BD-p53 + AD-T; Negative, BD-lam + AD-T and empty BD + AD-SITCP25. (B) LCI assay showing the influence of SlCycB2 on the interaction between Wo and SlTCP25. GFP-MYC was used as a negative control. (C) Trichome phenotype of AC, *Slcycb2* mutant, *Sltcp25* mutant, and the *Sltcp25 Slcycb2* double mutant. Bar, 500 μm. (D) The average branch number of the type I trichomes on hypocotyl of the indicated lines in (C). Data are means ± SD (n = 10). Significance differences were determined using one-way ANOVA.

Previous studies suggested that Wo interacts with SlCycB2 [24]. Thus, we tried to test whether SlCycB2 interacts with SlTCP25. However, Y2H assay showed that SlCycB2 did not interact with SlTCP25 in yeast (Fig. 7A). Since SlTCP25 and SlCycB2 separately interact with Wo, we inquired how SlCycB2 affects the interaction between Wo and SlTCP25. To test this possibility, we further performed LCI assay. When we co-expressed of SlTCP25-cLUC and SlCycB2-MYC with Wo-nLUC in *N. benthamiana*, there were no significant changes in LUC activity compared to the negative control (co-expression of SlTCP25-cLUC and GFP-MYC with Wo-nLUC) (Fig. 7B). These data suggest that SlCycB2 does not affect the interaction between Wo and SlTCP25.

To detect the genetic relationship between SlTCP25 and SlCycB2, we generated *Sltcp25 Slcycb2* double mutants by crossing. Compared to *Slcycb2* lines or *Sltcp25* lines, the trichomes exhibit more branches in *Sltcp25Slcycb2* double mutants (Fig. 7C and D). Taken together, these results suggest that SlTCP25 may interact indirectly with SlCycB2 to inhibit trichome branching in tomato.

### SlTCP25 weakens wo-regulated *SlCycB2* expression

Wo acts upstream of *SlCycB2* and promotes its expression [24, 27]. Since Wo interacts with SlTCP25, we further tested whether Wo and SlTCP25 could synergistically participate in the regulation of *SlCycB2* expression using transient transactivation assays. Then, we generated Wo and SlTCP25 effector constructs driven by the CaMV 35S promoter and the *GUS* gene reporter construct driven by the *SlCycB2* promoter (Fig. S8A). Compared to Wo expression alone, co-expression of Wo with SlTCP25 in *N. benthamiana* leaves can significantly reduce the *GUS* activity driven by the *SlCycB2* promoter. qRT-PCR analysis showed that *GUS* transcription level was reduced upon co-expression of both Wo and SlTCP25 compared to the control combinations, consistent with the results from the above *GUS* staining (Fig. S8B and C). Together, these data suggest that SlTCP25 interacts with Wo to alleviate Wo-regulated *SlCycB2* expression.

## Discussion

Trichomes have evolved different morphological characteristics. The complex structure of trichomes can physically or chemically prevent the invasion of pests. *Wo* acts as a positive regulator of type I trichome initiation [24]. However, the role of *Wo* in trichome branch formation remains unclear. In this study, we identified *Wo* as a positive regulator in trichome branch formation. Mutation in *Wo* and overexpression of *Wo* leads to the trichome branching on the hypocotyls (Fig. 1). However, knockout of *Wo* did not lead to branch formation on trichomes, suggesting that *Wo* may have a genetic compensation response with other unknown genes. Knockout of *Wo* also leads to an increase in the transcription level of *SlTCP25*. Therefore, these results may be the reason why knocking out Wo did not lead to the trichome branching. Since trichomes have very few branches on stems and leaves of LA3186 (Fig. S1), it is possible that there is a tissue specificity in trichome branch development. Combining previous reports, we confirmed that *Wo* not only regulates the initiation of type I trichomes, but also participates in the morphogenesis of type I trichomes in tomato.

The stalk cells of type I trichomes in *hl* mutant are twisted and swollen, which hinder vertical growth of type I trichome and cause overall structural distortion. Similar phenotypes were also observed in the *hl-2*, *hl-3* mutant, and *SIHDZIV8* RNAi lines in tomato [32–34]. However, branched trichomes maintain upright growth but not swelling or bending phenotypes in *Wo* mutants and *Wo-OE* lines, suggesting that the trichome branch formation controlled by Wo may be different from the previous mechanisms that has been reported in tomato. Previous studies showed the involvement of *TCP4*, *TCP14*, and *TCP15* in the regulation of trichome branch formation. *TCP14* and *TCP15* belong to the class I family, while *TCP4* belongs to the CIN subclass of class II family [40]. These genes only affect trichome branch number but not other morphological features in Arabidopsis [12, 14]. Unlike the said TCP family members in Arabidopsis, SlTCP22 and SlTCP25 belong to the CYC/TB1 subclass of class II family in tomato, and both are homologs of Arabidopsis TCP1 (Fig.S3B). Here, we found that trichomes produced many branches on the hypocotyls in *SlTCP25* knockout lines (Fig. 2C–G), similar to LA3186 and *Wo-OE* plants. However, there was a slight difference in the phenotype of branched trichomes in the *Sltcp25* lines and LA3186 plants. The branch points were located in the upper, middle, and bottom positions of trichome cells in LA3186, while the branch points were absent at bottom positions of trichome cells in the *Sltcp25* lines (Fig. S5A), suggesting that the initiation time of the trichome branches in the two mutants may be different. Genetic data analysis showed that *SlTCP25* is epistatic to *Wo* in branch trichome development (Fig. 4). Subsequently, biochemical experiments confirmed that Wo acts upstream of *SlTCP25* and inhibits its expression (Fig. 3). These results suggest that *Wo* may regulate the trichome branching in a pathway that partially overlaps with *SlTCP25*.

Phylogenetic analysis showed that SlTCP22 is highly similar to SlTCP25 (Fig. S3), and functional analysis showed that both SlTCP22 and SlTCP25 are involved in regulating the formation of trichome branching. The phenotype of the trichome branches is similar in the *Sltcp22* and *Sltcp25* lines. Most TCP transcription factors exhibit functional redundancy in regulating plant growth and development [13, 41, 42]. The *sltcp22sltcp25* double mutants exhibit significantly increased branch number of trichomes compared to *sltcp22* and *sltcp25* single mutants (Fig. 6A–C), suggesting that there is a functional redundancy between *SlTCP22* and *SlTCP25*. The increased branch number of the type I trichomes results in its abnormal development, a phenotype with reduced trichome height. However, the mechanism that *SlTCP22* and *SlTCP25* affect the development of trichomes by regulating branch number still needs to be explored in future studies. Biochemical experiments confirmed the physical interaction between SlTCP22 and SlTCP25 (Fig. 6D–F), further supporting that the two genes regulate trichome branching in a parallel pathway. There are 30 TCP genes that had been identified in tomato [43]. The TB1 subfamily of TCP contains six members, and further gene editing and cross experiments are necessary to create higher-order mutant so as to test whether the other TCPs exhibit functional redundancy in regulating trichome branching.

Cyclin and cyclin-dependent kinases are involved in cell division and endoreduplication, and TCP transcription factors can affect trichome branch formation by regulating the expression of cyclin and cyclin-dependent kinases [16, 18, 44, 45]. We previously identified one B-type cyclin, *SlCycB2*, as a negative regulator of trichome formation, while a recent study suggested that the cyclin may also function as E3 ubiquitin ligases [23, 25]. RPN1a, a component of the 26S proteasome, regulates nuclear endoreduplication processes in cells, resulting in an increase in the number of trichome branches in Arabidopsis [46, 47], suggesting that the branch formation of trichomes may also be related to the regulatory mechanism of ubiquitination. In this study, we discovered that the *Slcycb2* lines exhibited a branched trichome phenotype on the hypocotyls (Fig. S7), similar to that observed in *Sltcp25* lines. Therefore, we concluded that *SlTCP25* may act in the same pathway as *SlCycB2* during trichome branch formation. However, molecular experiments demonstrated SlTCP25 did not interact with SlCycB2 (Fig. 7A). Genetic data showed that the *Sltcp25Slcycb2* double mutants exhibited more trichome branches compared to *Sltcp25* or *Slcycb2* single mutant (Fig. 7C and D), suggesting that SlTCP25 may interact indirectly with SlCycB2 in regulating trichome branching. Whether the indirect interaction is mediated by Wo or other unknown genes remains to be elucidated. Moreover, the interaction between Wo and SlTCP25 can weaken the transcription level of *SlCycB2* (Fig. S8). However, whether *SlCycB2* functions as an E3 ubiquitin ligase or cyclin remains to be confirmed in future. In summary, these results implying that the Wo-SlTCP25 module regulates trichome branch formation by regulating the expression of *SlCycB2*. The model provides a new perspective on multicellular trichome morphogenesis and polarity establishment in plant.

## Materials and methods

### Plant materials and growth conditions

Tomato (*Solanum lycopersicum*) seedlings and tobacco (*N. benthamiana*) plants were grown in a growth chamber under standard conditions (16-hour light and 8-hour dark). Four-week-old *N*. *benthamiana* plants were chosen for subsequent injection assays. Ailsa Craig (AC) and *Wo* allelic mutants (LA3186 and LA3560) were used in this study. AC was used as a wild type and for generating transgenic plants in this research.

### Vector construction and plant transformation

To generate *SlTCP22, SlTCP25*, and *SlCycB2* knockout constructs, we designed two target sites for each gene and inserted them into p201N Cas9 vector. These target sites were designed using the CRISPR2.0 website (CRISPR-P v2.0 (hzau.edu.cn)). For overexpression constructs, the full-length coding sequence (CDS) of *Wo* was amplified from LA3186 and inserted into the pHellsgate8 vector with CaMV 35S promoter. The *Agrobacterium strain* GV3101 (WEIDI, Shanghai, China) containing the recombinant plasmids were introduced into AC background. Primers used in this study are listed in Table S1.

### Trichome phenotypic characterization

Tomato trichome samples were collected from 3-week-old hypocotyls, stems, and leaflets for stereomicroscopy analysis (Leica M205FA, Germany). Three-week-old hypocotyls of tomato plants were collected for cryo-scanning electron microscope (Cryo-SEM) (Hitachi SU8010, Japan) analysis. Images were obtained and assembled using Adobe Illustrator 2022 software.

### Subcellular localization

To investigate the subcellular localization of SlTCP25, the full-length CDS of *SlTCP25* were cloned into the 35S: YFP fusion vector. Empty 35S: YFP and nuclear marker 35S-NLS: RFP constructs were used as controls. The *Agrobacterium strain* containing 35S:*SlTCP25*-YFP fusion constructs and the controls were co-infiltrated into four-week-old *N. benthamiana* leaves. The *N. benthamiana* plants were incubated under low light conditions for 2d. Fluorescent images were captured using a confocal microscope (Leica SP8, Germany).

### RNA isolation and expression analysis

Total RNA was extracted from different tissues using a TRIzol reagent Kit (Aidlab, Beijing, China). Subsequently, first-strand cDNA was synthesized with the HiScript II 1st Strand cDNA Synthesis Kit (Vazyme, Nanjing, China) and identified by a pair of specific actin primers. Quantitative real-time PCR (qRT-PCR) was carried out to detect the transcript levels of genes on a QuantStudio3 detection system (ThermoFisher Scientific, China). The tomato actin gene (Solyc11g008430) was used as an internal control. The data analysis was performed from three biological and three technical replicates.

### Phylogenetic analysis and multiple sequence alignment

The amino acid sequences of TB1-subfamily transcription factors from *S. lycopersicum*, *Oryza sativa*, and *Arabidopsis* were obtained from the Phytozome website (https://planttfdb.gao-lab.org/). Multiple alignments were performed by Clustal W. Phylogenetic trees were constructed using the Neighbor-joining method with 1000 bootstrap replicates in MEGA11 software.

### GUS histochemical staining

The *N. benthamiana* leaves infiltrated by *Agrobacterium strain* were immersed in GUS staining solution and then were incubated overnight at 37°C, followed by decolorization with 75% alcohol for 24 hours. Subsequently, the leaves were photographed for further observation.

### Y1H assay

The full-length CDS of *Wo* from LA3186 was amplified and cloned into the pGADT7 vector. The *SlTCP25* promoter sequence containing the L1-box *cis-*element was cloned into the pAbAi vector to generate the pAbAi-*SlTCP25* construct. The pAbAi-*SlTCP25* recombinant plasmid was linearized using the *BstBI* restriction endonuclease (NEB, England). Following the manufacturer’s instructions, the yeast strain Y1H Gold was transformed with the linearized pAbAi-*SlTCP25* construct and selected on SD/-Ura medium. These yeast strains containing pAbAi-*SlTCP25* plasmid was then transformed with pGADT7-Wo plasmid and selected on SD/−Leu medium with different Aureobasidin A (AbA) concentrations. An empty pGADT7 vector was used as a negative control.

### Dual-luciferase reporter assay

The *Wo* full-length CDS was cloned into the pGreenII 62-SK effector vector, and the 2.5 kb promoter fragment of *SlTCP25* was cloned into the pGreenII 0800-LUC reporter vector. These recombinant plasmids were then introduced into *Agrobacterium strain* GV3101 with the helper pSoup19 plasmid. Then, these *Agrobacterium strains* were co-infiltrated into one-month-old *N. benthamiana* leaves. Two days after infiltration, samples were collected and analyzed using a Dual-Luciferase Reporter Gene Assay Kit (YEASEN, Shanghai, China). The activity values of firefly luciferase and renilla luciferase were measured using a Glomax device (Promega, USA).

### Electrophoretic mobility shift assay

The full-length CDS of *Wo* was cloned into the pET-15d vector with an MBP tag to generate the Wo-MBP construct, and the recombinant plasmid then was transformed into BL21 strain (TOLOBIO, Shanghai, China). Protein expression was induced using 0.5 mM IPTG and purified using a His-Tagged Protein Purification Kit (CWBIO, Jiangsu, China). The probe containing L1-box element from *SlTCP25* promoter was synthesized by Tsingke (Beijing, China) with 5’ FAM. The reaction between the target protein and the probe was carried out according to the EMSA/Gel-Shift manual instruction (Beyotime, Shanghai, China). In brief, the target protein was firstly allowed to react with cold probes for 10 minutes at room temperature, and the labeled probes were then incubated with the above system for 20 minutes at room temperature. The loading buffer was added to the reaction system, and the samples were immediately loaded into polyacrylamide gel. The result was observed using a Fluor Chem M system (Protein Simple, San Jose, CA, USA).

### Y2H assay

The full-length CDS of *Wo* and *SlTCP25* was respectively amplified and cloned into the pGADT7 and pGBKT7 vector to yield pGADT7-Wo and pGBKT7-SlTCP25 construct. Likewise, the full-length CDS of *SlTCP25* and *SlCycB2* were cloned into the pGADT7 vector, respectively. These recombinant plasmids were co-transformed into the AH109 yeast strain. The yeast transformants were selected on SD/−Leu-Trp and SD/−Leu-Trp-His-Ade agar medium at 30°C for 3d. pGBKT7-P53 was used as a positive control. pGBKT7-lam, empty pGBKT7, and empty pGADT7 were used as negative controls.

### Bimolecular fluorescence complementation

The full-length CDS of *Wo* and *SlTCP25* were amplified and inserted into pSPYNE173 and pSPYCE155 to yield SlTCP25-NE and Wo-CE constructs, respectively. An empty pSPYCE155 vector was used as a negative control. The *Agrobacterium strain* was transformed with the resulting plasmids and then infiltrated into one-month-old tobacco leaves. Two days after infiltration, tobacco leaves were collected for observation. The YFP fluorescence signals were visualized using a confocal microscope.

### Luciferase complementation imaging

For the LCI assay, the CDS of *Wo* and *SlTCP25* was amplified and respectively inserted into the pCAMBIA-nLUC/cLUC vector to generate Wo-nLUC and SlTCP25-cLUC constructs. Empty n-LUC and c-LUC vector were used as negative controls. The *Agrobacterium strain* containing Wo-nLUC and SlTCP25-cLUC plasmids were co-infiltrated into tobacco leaves. After 2d of infiltration, the leaves abaxial were coated with 1 mM D-Luciferin potassium salt for further observation. The luciferase fluorescence images were captured using a plant living imaging system (Berthold, Night Shade LB 985).

### Co-IP assay

The full-length CDS of *SlTCP22* and *SlTCP25* were cloned into PH7LIC vector with GFP or MYC tag, respectively. The recombinant plasmids were introduced into the *Agrobacterium strain* for the co-immunoprecipitation assay. Healthy one-month-old tobacco leaves were chosen for injection. After 2d of infiltration, the tobacco leaves were collected, and total protein was extracted for an input assay to assess the target protein expression. Tobacco total protein and anti-MYC agarose beads were co-incubated and immunoprecipitated using GFP and MYC antibodies, respectively. The result of co-immunoprecipitation was detected using an ECL chemiluminescence reagent.

## Supplementary Material

Web_Material_uhaf032

## Data Availability

The data that supports the findings of this study are available in the supplemental information of this article.
