# Supplementary material for: Wo interacts with SlTCP25 to regulate type I trichome branching in tomato
Source: Hortic Res. 2025 Jan 5;12(5):uhaf032. doi: 10.1093/hr/uhaf032 (PMC11992337; doi:10.1093/hr/uhaf032)
Supplement: Web_Material_uhaf032 [file web_material_uhaf032.zip › Supplemental information(clean).docx]

## Horticulture research

**Article title:** Wo interacts with SlTCP25 to regulate type I trichome branching in tomato

**Authors:** Junqiang Wang^1^, Shoujuan Yuan^1^, Yihao Zhao^1^, Xin Shu^1^, Lin Wang^1^, Zhiling Liu^1^, Taotao Wang^1^, Zhibiao Ye^1^, Changxian Yang^1*^

The following Supporting Information is available for this article:

**Supplemental Figures:**

**Figure S1.** Morphology of trichomes on the stem and leaf of AC and LA3186.

**Figure S2.** Phenotypic analysis of *Wo* allele mutant.

**Figure S3.** Multiple sequence alignment and phylogenetic tree analysis of TCP subfamily CYC/TB1-type genes.

**Figure S4.** Expression patterns of *SlTCP25* in different tissues.

**Figure S5.** Trichome branch location in the *Sltcp25* lines.

**Figure S6.** Knockout of *SlTCP22* led to the formation of branch trichomes in tomato.

**Figure S7.** *SlCycB2* as a negative regulator in trichome branch formation.

**Figure S8.** SlTCP25 alleviate Wo-regulated *SlCycB2* expression.

**Supplemental Tables:**

**Table S1** Sequences of primers used in the research.


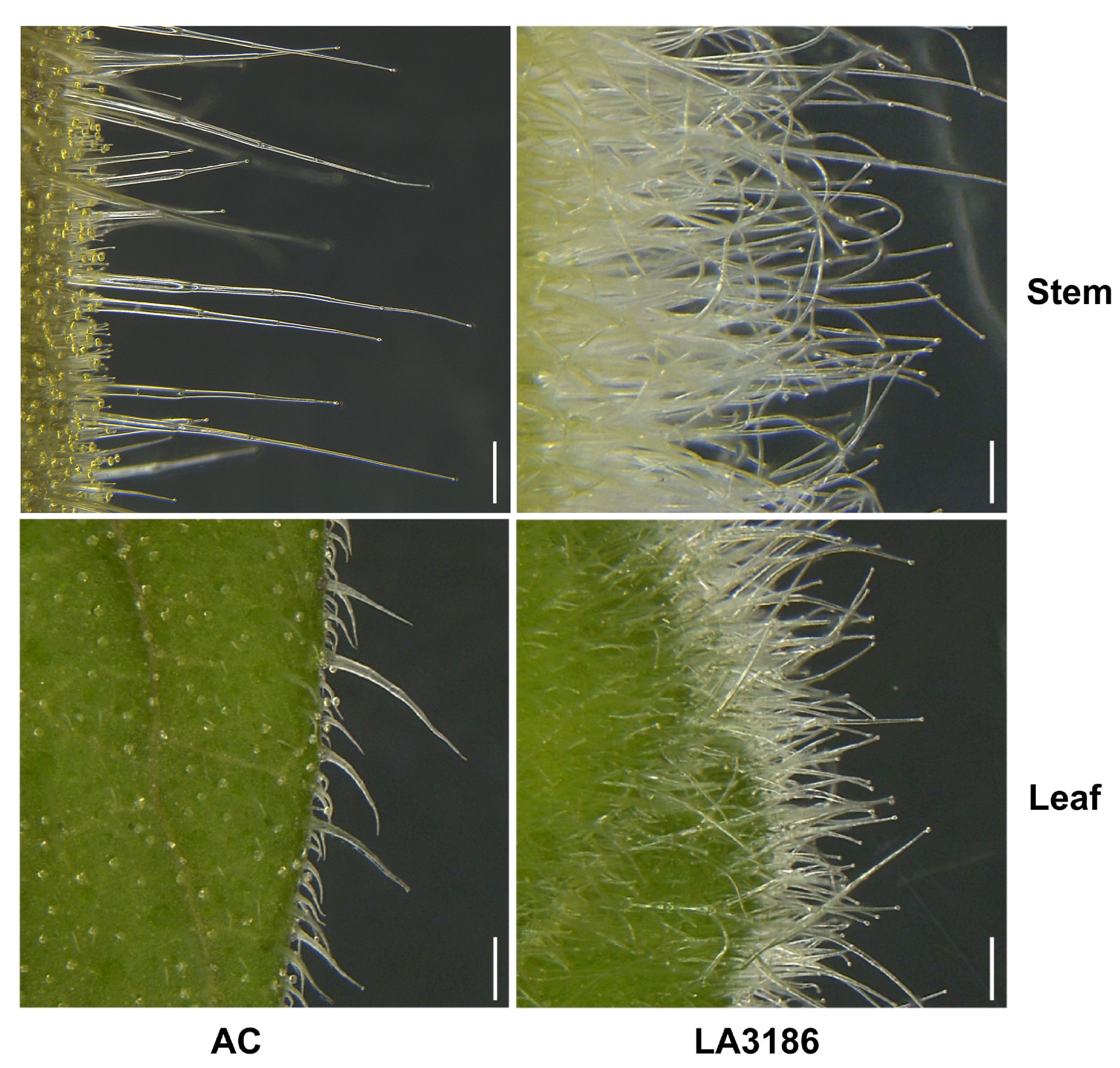


**Fig.S1 Morphology of trichomes on the stem and leaf of AC and LA3186**.

The images are present here from stereomicroscope. The stems and leave are taken from three-week-old tomato plants. Bar, 200µm.


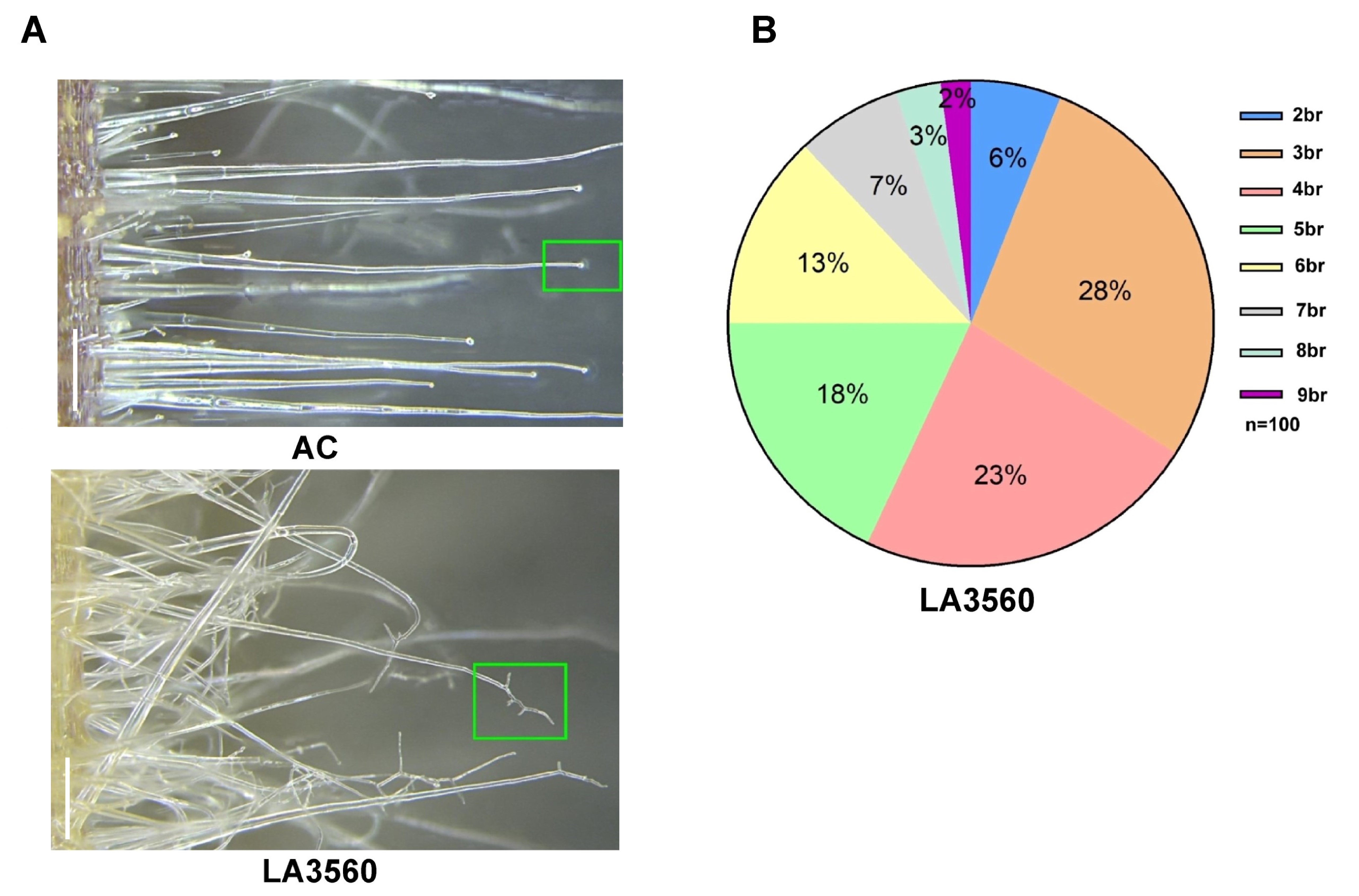


**Fig.S2 Phenotypic analysis of *Wo* allele mutant**.

**(A)** Morphology of trichomes in AC and LA3560. Three-week-old hypocotyl was taken for investigation. Bar, 500µm. **(B)** The branch percentage of type I trichomes on the hypocotyl of LA3560 plants (n=100). br, branch number of per trichome.


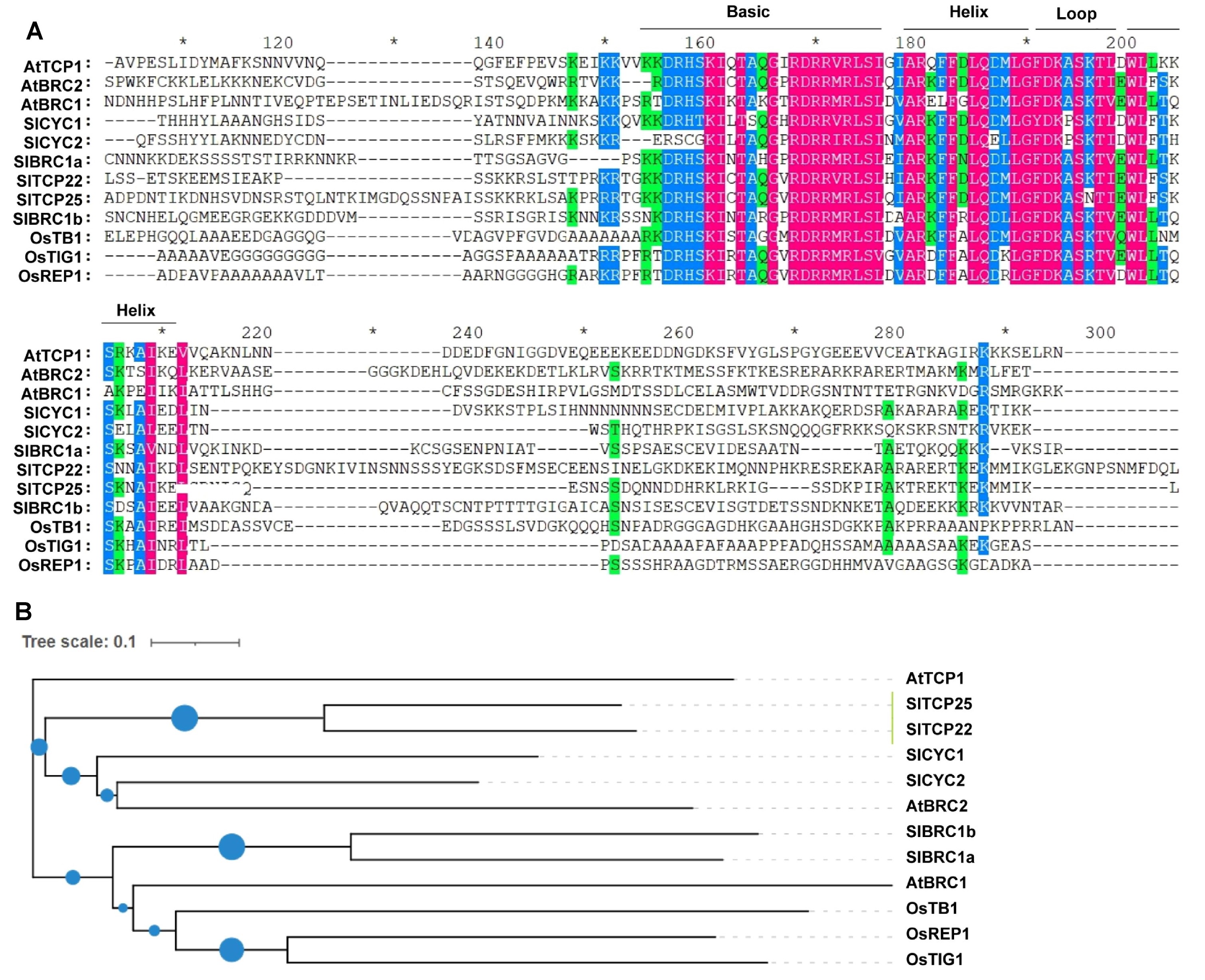


**Fig.S3 Multiple sequence alignment and phylogenetic tree analysis of TCP subfamily CYC/TB1-type genes**.

**(A)** Multiple sequence alignment of the homologs of *SlTCP25*. Conserved bHLH domains are represented by colored letters. **(B)** Phylogenetic tree was constructed using the homologs of *SlTCP25* from *Arabidopsis thaliana* (*At*), *Oryza sativa* (*Os*) and *Solanum lycopersicum* (*Sl*).


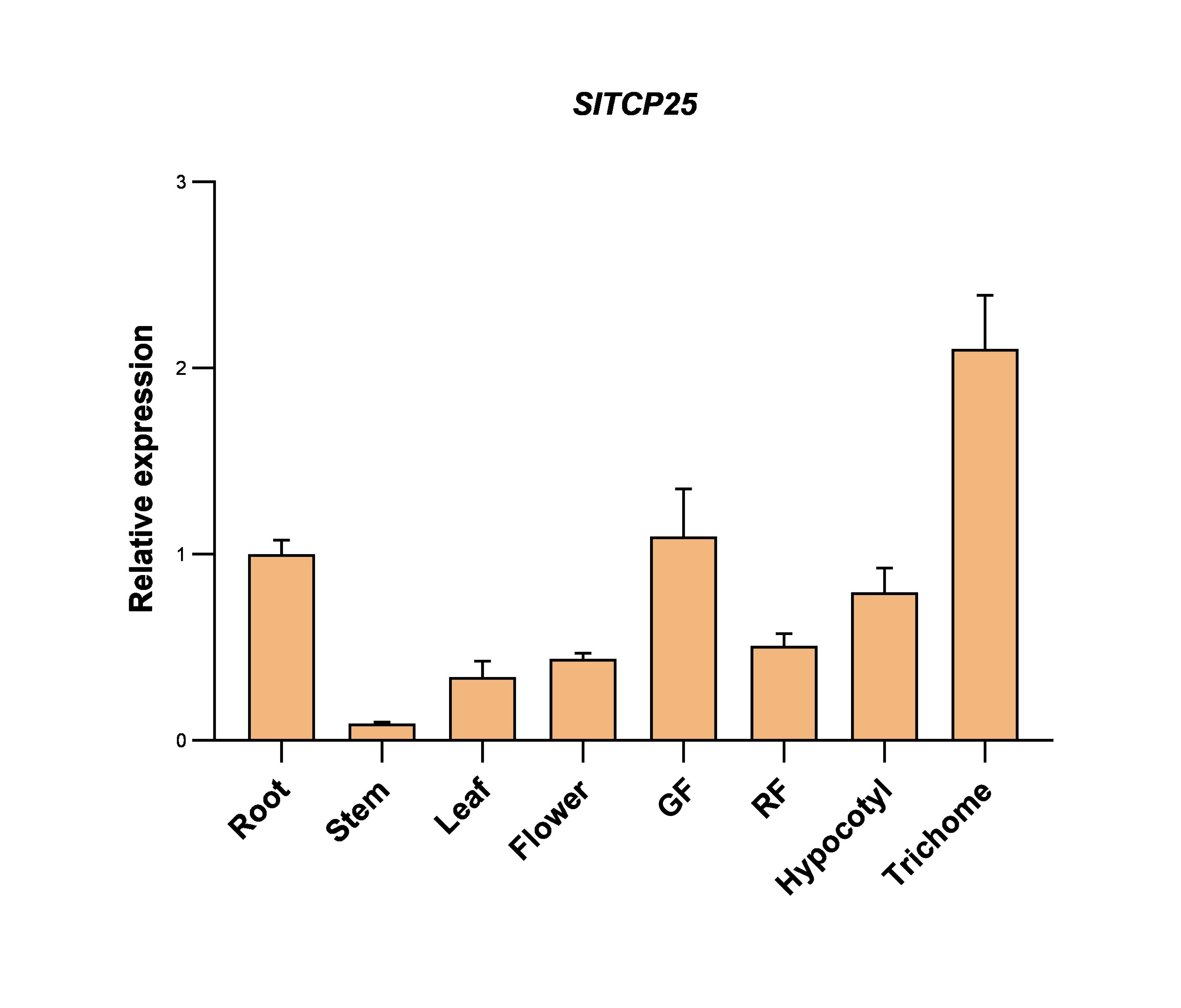


**Figure S4. Expression patterns of *SlTCP25* in different tissues.**

RNA samples were collected from Root, Stem, Leaf, Flower, GF (green fruit), RF (red fruit), hypocotyl, and trichomes in tomato. The expression data were determined using qRT-PCR. Data are means ± SD (n = 3).


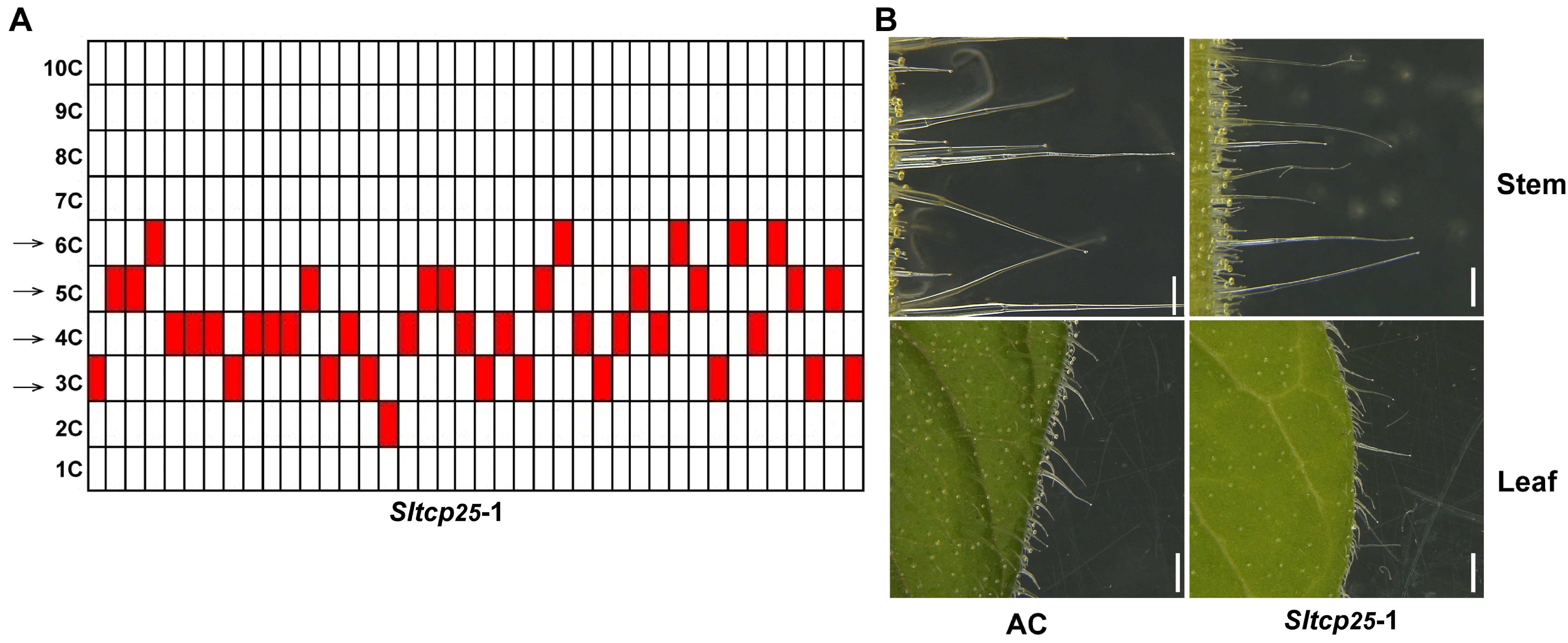


**Fig.S5 Trichome branch location of the *Sltcp25* lines.**

**(A)** Branch occurs on the trichomes of the *Sltcp25* lines at positions 1-10c, where “c” indicates cell position from bottom to top of type I trichomes. The white boxes represent non-branch cells, and red boxes represent branch cells (n=60). **(B)** Morphology of trichomes on the stem and leaf of AC and the *Sltcp25* lines. The samples were taken from three-week-old plants. Bar, 200µm.


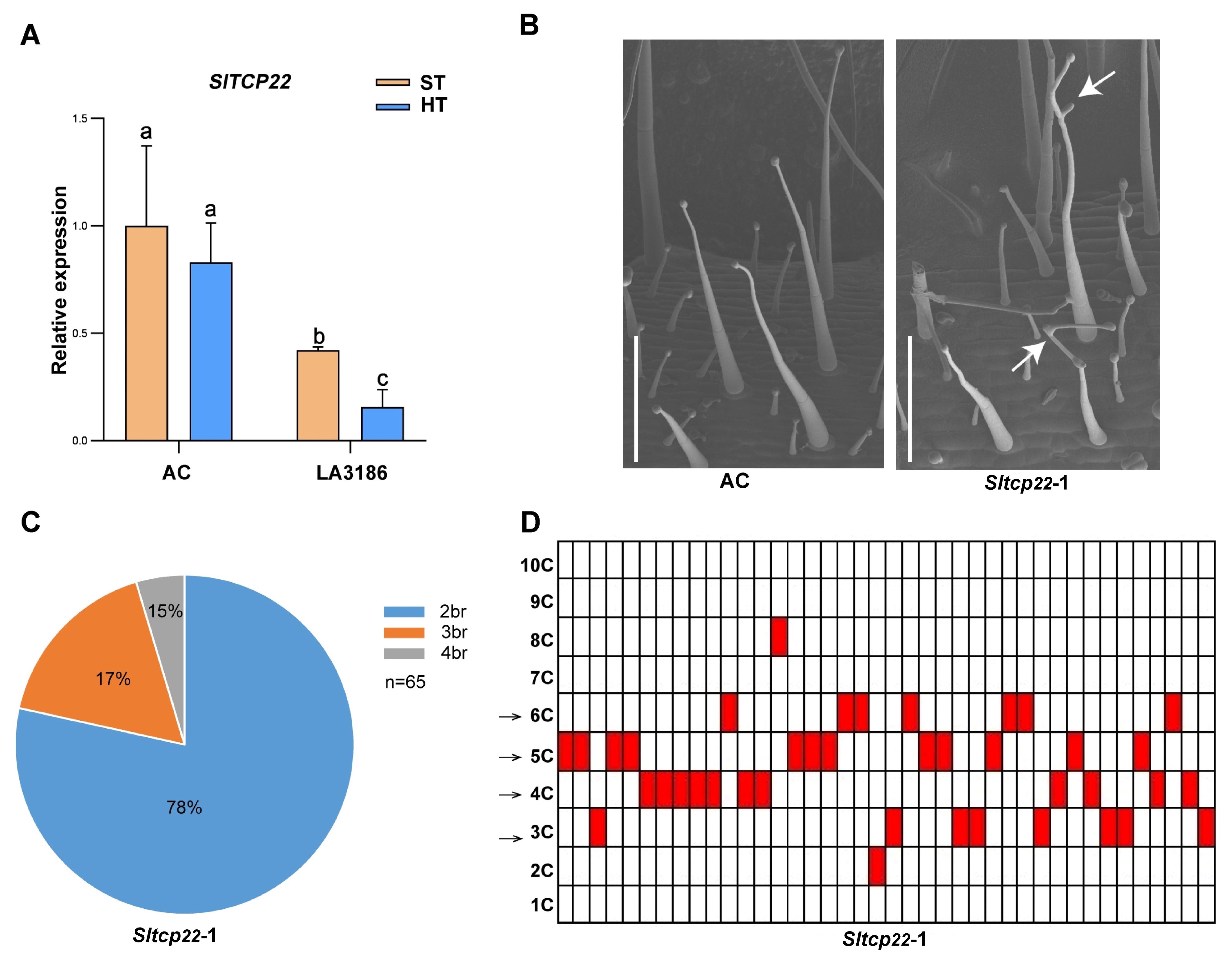


**Fig.S6 Knockout of *SlTCP22* led to the formation of branch trichomes in tomato.**

**(A)** Expression levels of *SlTCP22* in AC and LA3186 plants. ST, stem trichomes; HT, hypocotyl trichomes. Values are given as means ± SD (n = 3). Actin was used as an internal reference. Significance analysis was performed using one-way ANOVA, where distinct letters indicate significant differences. **(B)** The images of trichome phenotype on the hypocotyl of AC and the *Sltcp22* lines from Cryo-scanning electron microscopy. Bar, 500µm. **(C)** The branch percentage of the type I trichomes on the hypocotyl of the *Sltcp22* lines. br, branch number per trichome (n=65). **(D)** Branch locations on trichome cells of the *Sltcp22* lines. Branch occurs on the trichomes at positions 1-10c, where “c” indicates cell position from bottom to top of type I trichomes. The white boxes represent non-branch cells, and red boxes represent branch cells (n=60).


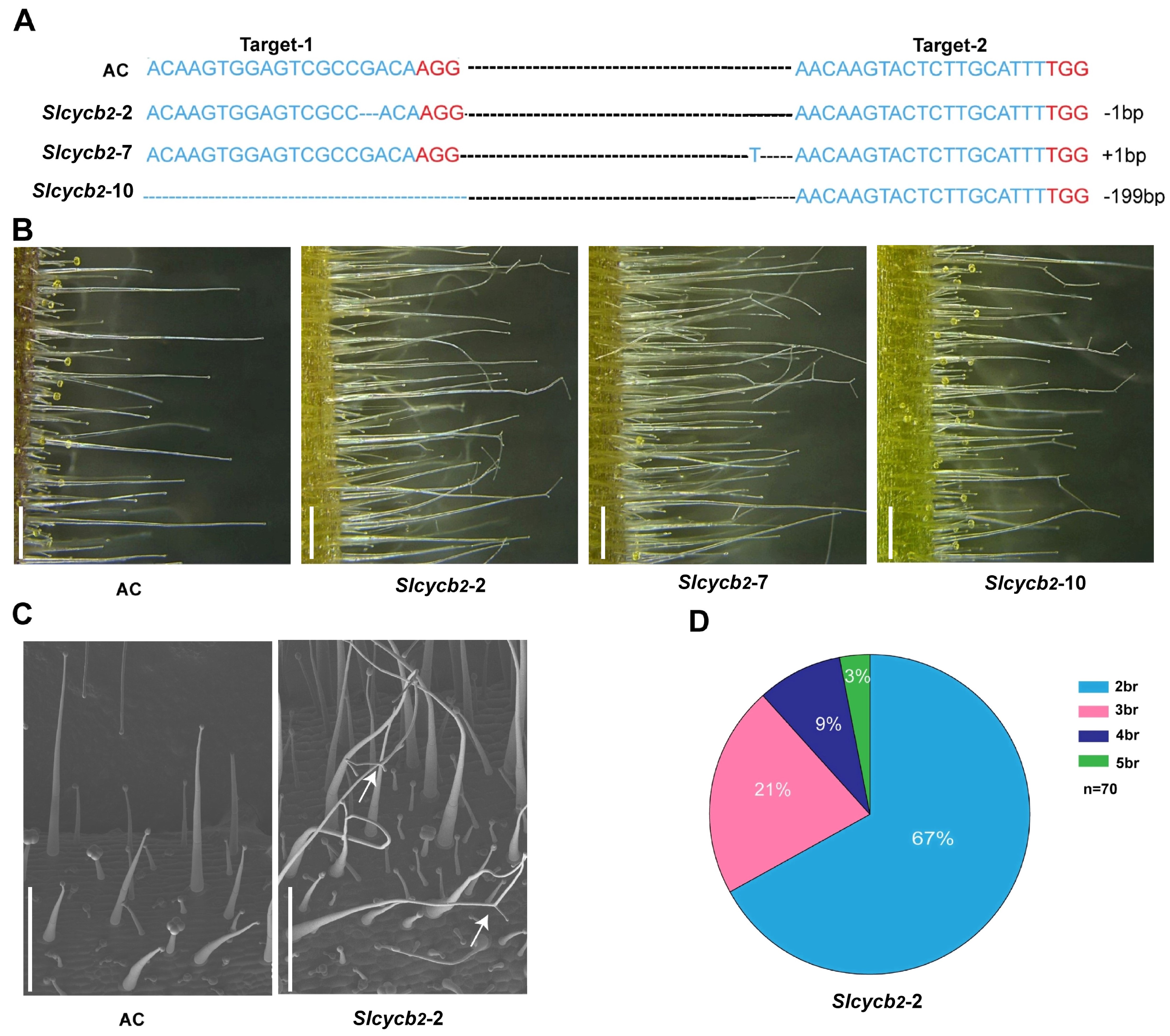


**Fig.S7 *SlCycB2* as a negative regulator in trichome branch formation.**

**(A)** The gene editing results of the *SlCycB2* knockout lines identified by sequencing. The red letters represent the PAM site, and the right number of the target indicates the inserted or missing base, which caused frameshift mutation. **(B) and (C)** Hypocotyl trichomes of the *Slcycb2* lines. The samples were collected from three-week-old plants. Bar, 500µm. **(D)** The branch percentage of type I trichomes on hypocotyl of the *Slcycb2* lines (n=70).

***
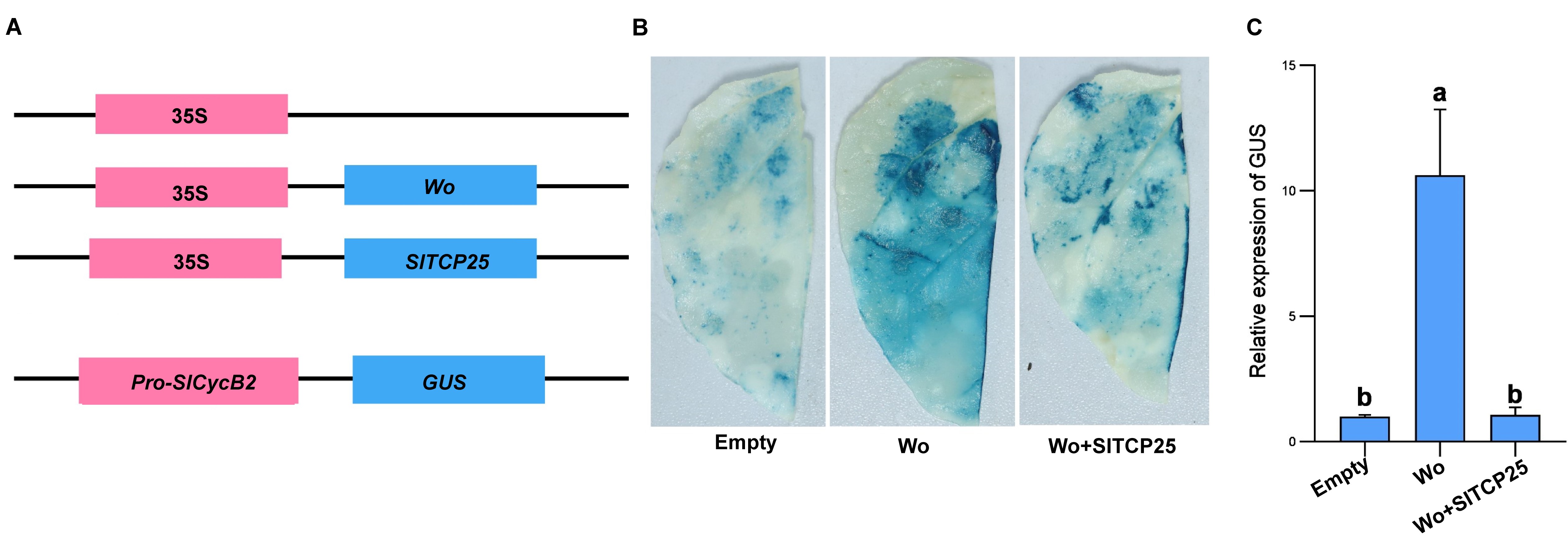
***

**Figure S8. SlTCP25 alleviate Wo-regulated *SlCycB2* expression.**

**(A)** Schematic representation of effector construct and reporter construct. The *GUS* reporter construct was driven by the *SlCycB2* promoter. **(B)** *GUS* staining in *N. benthamiana* leaves after co-expression of effector with reporter gene. Empty construct driven by 35S promoter was used a negative control. **(C)** The expression levels of *GUS* in *N. benthamiana* leaves. Data are means ± SD (n = 3). Statistically significant differences were determined using one-way ANOVA.

**Table S1** Sequences of primers used in the research.

| **Primers** | **Sequence (5'-3')** | | |  |  |  |
| --- | --- | --- | --- | --- | --- | --- |
| *SlTCP22*-Q | AAGAGGACGGGGAAGAAGGATA | | | | |  |
|  | GGAGATCAAAGAACTTACGCGC | | | | |  |
| *SlTCP25*-Q | AGCAACATCATCCATGACCCTT | | | | |  |
|  | TGTGTTGTCTGGATCAGCTTGA | | | | |  |
| *GUS*-Q | AAGTGTCGGTCGTGGATGAG | | | | |  |
|  | AGTCCGTCGTTCACCAGTTC | | | | |  |
| *SITCP25*-gRNA | GAATCTAACAGTGTAGTTTGGTCATCAAGAAATGGAGATGTTTTAGAGCTAGAAATAGC | | | | |  |
|  | GAATCTAACAGTGTAGTTTGAAGTGCAAAACCGCGAAGGGTTTTAGAGCTAGAAATAGC | | | | | |
| *SITCP22*-gRNA | GAATCTAACAGTGTAGTTTGCGAAAGAGGACGGGGAAGAGTTTTAGAGCTAGAAATAGC | | |  |  |  |
|  | GAATCTAACAGTGTAGTTTGAGGGAAAAAGCAAGAGCAAGTTTTAGAGCTAGAAATAGC | | |  |  |  |
| *SlCycB2*-gRNA | GAATCTAACAGTGTAGTTTGACAAGTGGAGTCGCCGACAGTTTTAGAGCTAGAAATAGC | | | |  |  |
|  | GAATCTAACAGTGTAGTTTGAACAAGTACTCTTGCATTTGTTTTAGAGCTAGAAATAGC | |  |  |  |  |
| 35S:Wo-3× Flag | GATGACGATGACAAGGAATTCATGTTTAATAACCACCAGCACTTGC |  |  |  |  |  |
|  | GATGACGATGACAAGGAATTCTGCATTTGCGGAAGTTACAGCACC |  |  |  |  |  |
| 35S:SITCP25 | GATGACGATGACAAGGAATTCATGTTTCCTAAAAGCAACATCATCC |  |  |  |  |  |
| -3×Flag | GTCCTTGTAATCCATGAATTCCTAAAATTGATGAAACCTTGAA |  |  |  |  |  |
| 35S:SITCP22 | GATGACGATGACAAGGAATTCATGTTTCCTTCAAGCAATAACC |  |  |  |  |  |
| -3×Flag | GTCCTTGTAATCCATGAATTCTTCCAATCTATGATGTTTGCTGGATAG |  |  |  |  |  |
| pAbAi-*SITCP25* | AGCTTGAATTCGAGCTCGGTACCGAGGCAATTCAATAATGACC |  |  |  |  |  |
| (L1-box) | ACATACAGAGCACATGCCTCGAGGGGAGATAGTTCGGATGCAT |  |  |  |  |  |
| SITCP25-YFP | ATATGGGATCTACTAGTGAATTCATGTTTCCTAAAAGCAACATCATCC |  |  |  |  |  |
|  | TCGAGCCCGGGGGTACCGTCGACAAATTGATGAAACCTTGAA |  |  |  |  |  |
| 62SK-Wo | GCCGCTCTAGAACTAGTGGATCCATGTTTAATAACCACCAGCACTTGC |  |  |  |  |  |
|  | TTGGTACCGGGCCCCCCCTCGAGTCATGCATTTGCGGAAGTTACAGCACC |  |  |  |  |  |
| 0800-*SlTCP25* | CACTATAGGGCGAATTGGGTACCGTCGACACAAGGATACATTCATTGTAC |  |  |  |  |  |
|  | TATGTTTTTGGCGTCTTCCATGGATATAGGGTTTTGTAAGAAGAAC |  |  |  |  |  |
| *SlTCP25*- | TGCATCCAACGCGTTGGGAGCTCGTCGACACAAGGATACATTCATTGTAC |  |  |  |  |  |
| *pro-GUS* | GCCTTCGCCATTCTAGACTCGAGATATAGGGTTTTGTAAGAAGAAC |  |  |  |  |  |
| AD-Wo | ACGTACCAGATTACGCT CATATGATGTTTAATAACCACCAGCACTTGC |  |  |  |  |  |
|  | TACGATTCATCTGCAGCTCGAGCTCATGCATTTGCGGAAGTTACAGC |  |  |  |  |  |
| AD-SlTCP25 | ACGTACCAGATTACGCTCATATGATGTTTCCTAAAAGCAACATCATCC |  |  |  |  |  |
|  | TACGATTCATCTGCAGCTCGAGCCTAAAATTGATGAAACCTTGAA |  |  |  |  |  |
| BD-SlTCP25-2 | TCTCAGAGGAGGACCTGCATATGATGTTTCCTAAAAGCAACATCATCC |  |  |  |  |  |
|  | TTATGCGGCCGCTGCAGGTCGACGCTAAAATTGATGAAACCTTGAA |  |  |  |  |  |
| BD-SlTCP22 | TCTCAGAGGAGGACCTGCATATGATGTTTCCTTCAAGCAATAACC |  |  |  |  |  |
|  | TTATGCGGCCGCTGCAGGTCGACGTTATTCCAATCTATGATGTTTGC |  |  |  |  |  |
| BD-SlCycB2 | TCTCAGAGGAGGACCTGCATATG ATGAGCAGGAGAAATGGAAATG |  |  |  |  |  |
| SlTCP25-NE  Wo-CE | TTATGCGGCCGCTGCAGGTCGACG TTATTTTTTCTCATAGATAAC  GGCCCAGGCCTACTAGTGGATCCATGTTTCCTAAAAGCAACATCATC  AGCTCCTACCCGGGAGCGGTACCCTAAAATTGATGAAACCTTGAA  AGAGAACACGGGGGACTCTAGAATGTTTAATAACCACCAGCACTTGC  ATCCCGGGAGCGGTACCCTCGAGTGCATTTGCGGAAGTTACAGCACC |  |  |  |  |  |

| **Primers** | **Sequence (5'-3')** |  |
| --- | --- | --- |
| Wo-nLUC | CGAGCTCGGTACCCGGGATCCATGTTTAATAACCACCAGCACTTGC | |
|  | CGCGTACGAGATCTGGTCGACTCATGCATTTGCGGAAGTTACAGC | |
| SlTCP25-nLUC | CGAGCTCGGTACCCGGGATCCATGTTTCCTAAAAGCAACATCATCC | |
|  | CGCGTACGAGATCTGGTCGACCTAAAATTGATGAAACCTTGAA | |
| SlTCP25-cLUC | TACGCGTCCCGGGGCGGTACCATGTTTCCTAAAAGCAACATCATCC | |
|  | ACGAAAGCTCTGCAGGTCGACAAATTGATGAAACCTTGAA | |
| SlTCP22-cLUC | TACGCGTCCCGGGGCGGTACCATGTTTCCTTCAAGCAATAACC | |
|  | ACGAAAGCTCTGCAGGTCGACTTCCAATCTATGATGTTTGC | |
| SlTCP25-GFP | CGCTCGAGTCCGGAGGATCCATGTTTCCTAAAAGCAACATCATCC | |
|  | TCGCCCTTGCTCACTCTAGACTAAAATTGATGAAACCTTGAA | |
| SlCycB2-MYC | ATTACGCCGAGGTCATGAGCAGGAGAAATGGAAATGGTTC | |
|  | TAGGGAAGAGGTTATTTTTTCTCATAGATAACTTCAAGC | |
| Wo-MBP | TCTGTTCCAGGGGCCGCATATGATGTTTAATAACCACCAGCACTTGC | |
|  | TGTTAGCAGCCGGATCCTCGAGTCATGCATTTGCGGAAGTTACAGCACC | |
| *SlTCP25*-probe | CATAAAAGGTATGGTAAATGCTATCAGTATATAT | |
|  | ATATATACTGATAGCATTTACCATACCTTTTATG | |
