# Supplementary figures and images for: Wo interacts with SlTCP25 to regulate type I trichome branching in tomato
Source: Hortic Res. 2025 Jan 5;12(5):uhaf032. doi: 10.1093/hr/uhaf032 (PMC11992337; doi:10.1093/hr/uhaf032)

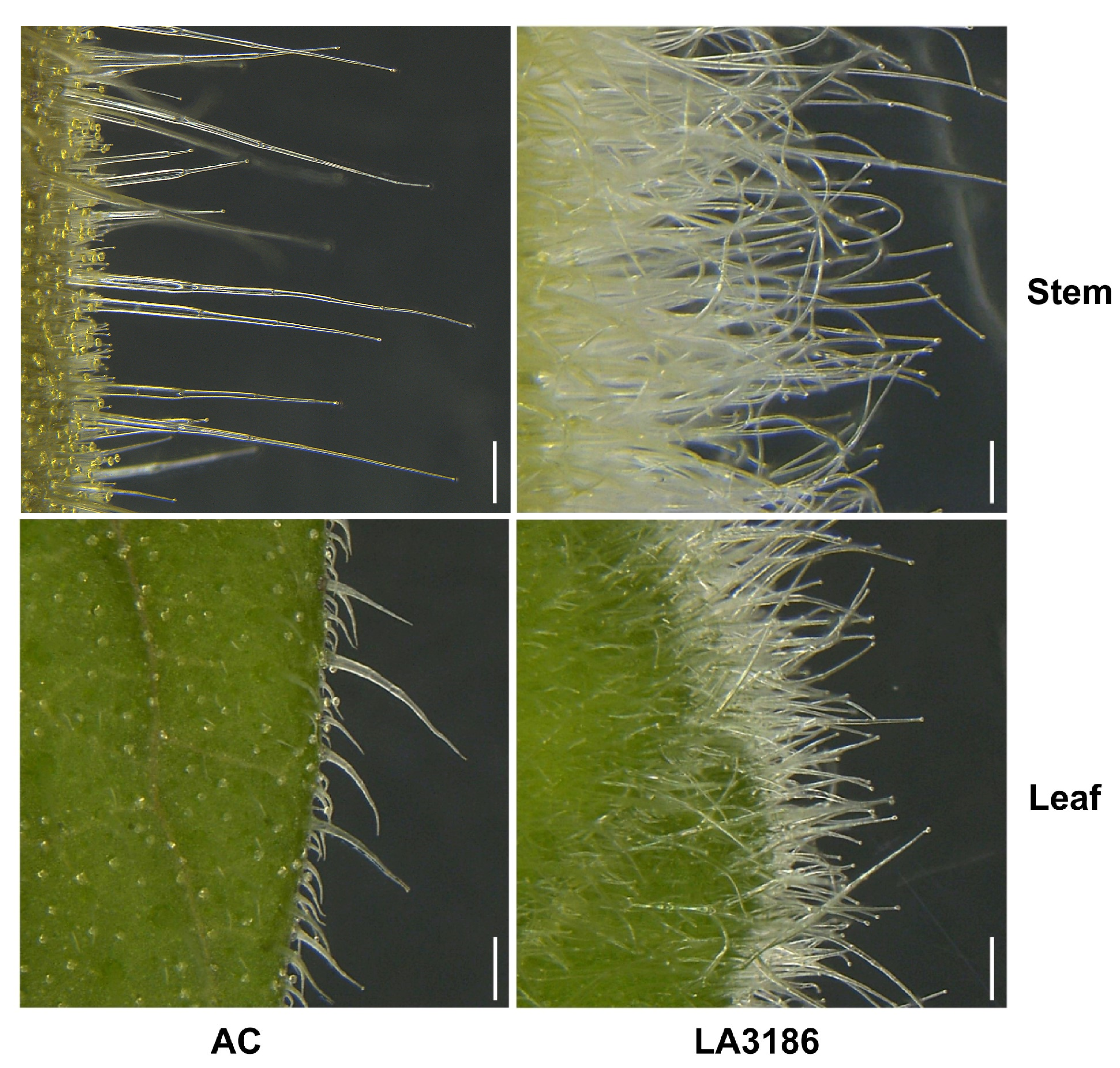

Supplement: Web_Material_uhaf032 [file web_material_uhaf032.zip › Figure S1.tif]

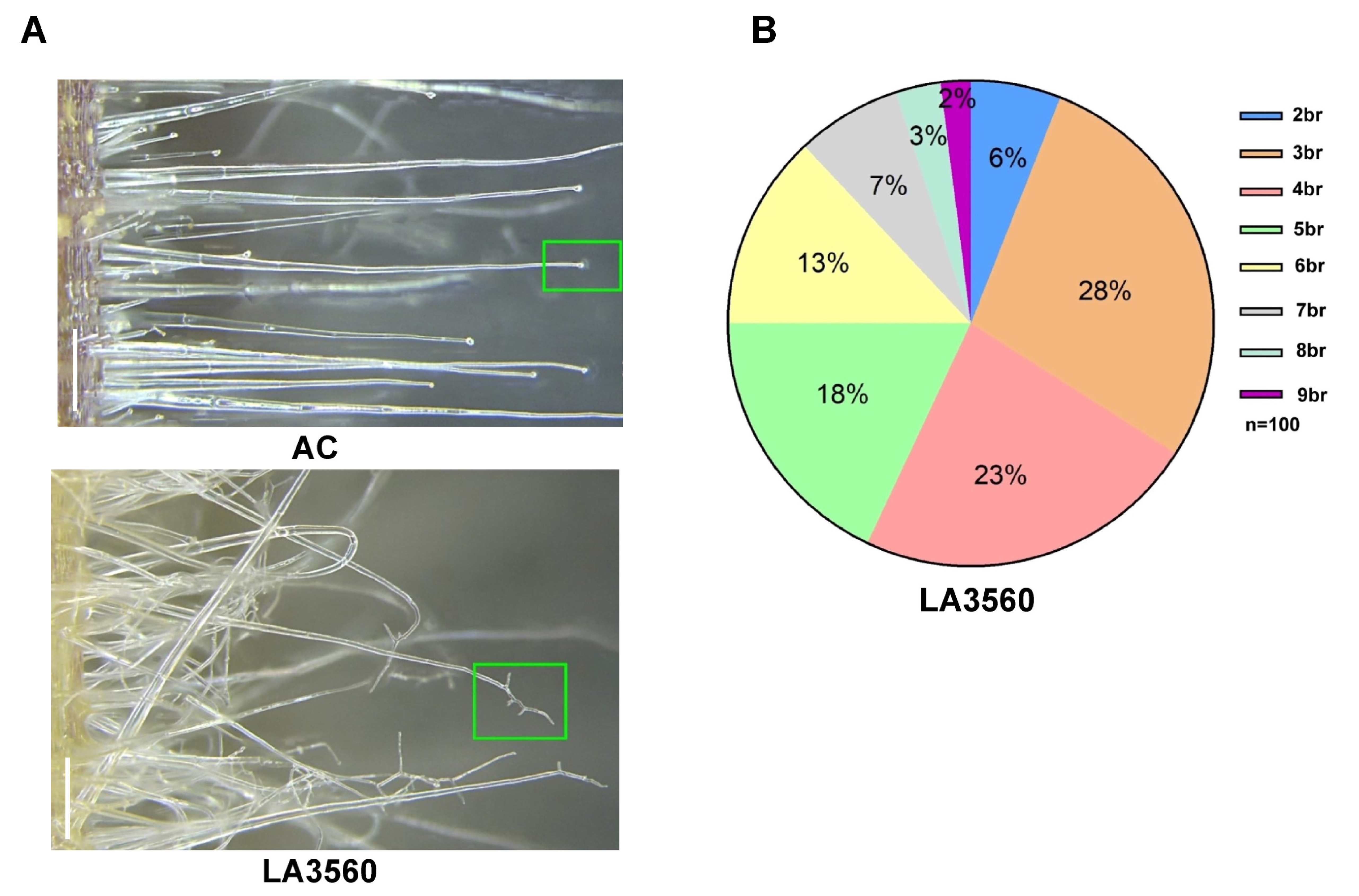

Supplement: Web_Material_uhaf032 [file web_material_uhaf032.zip › Figure S2.tif]

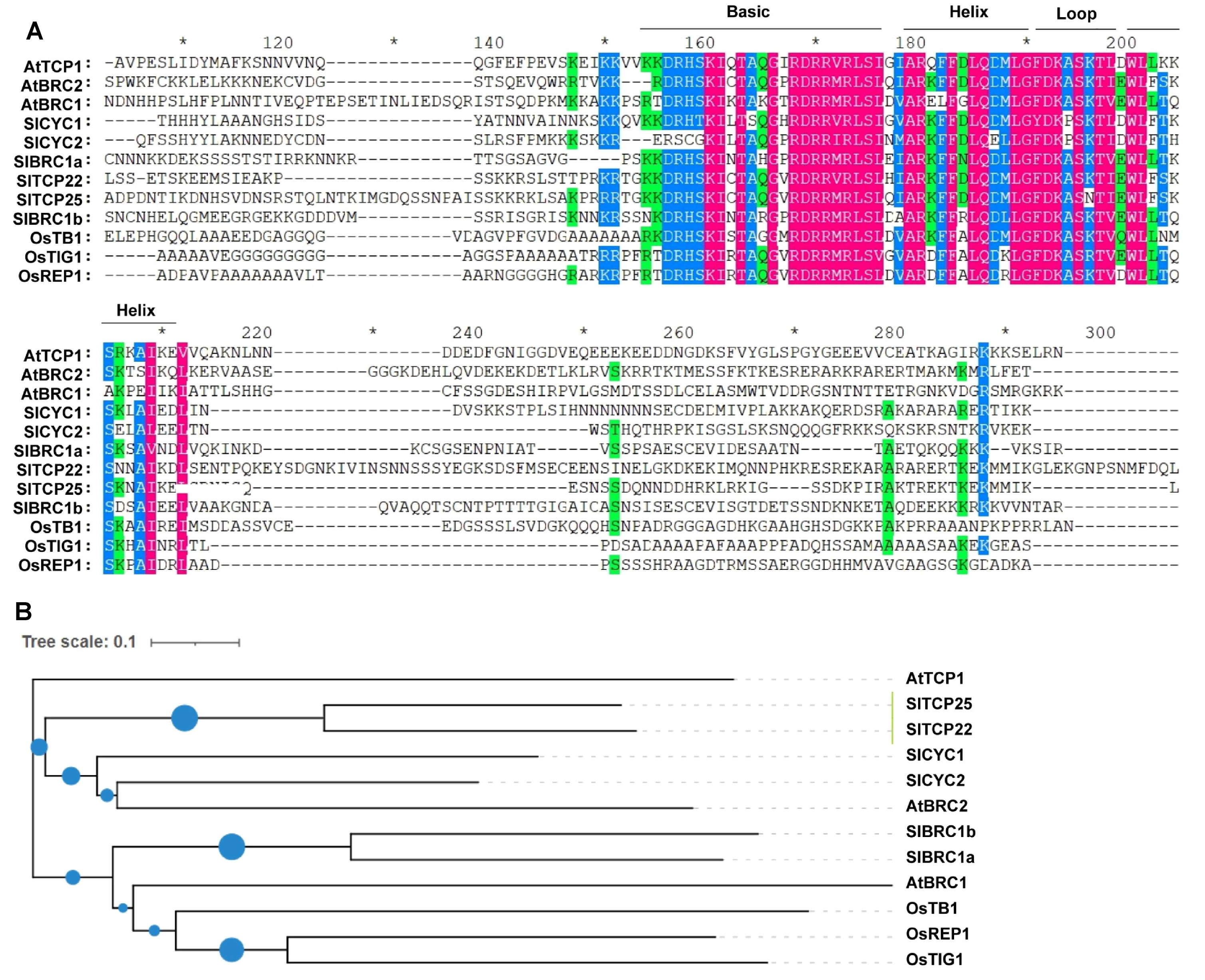

Supplement: Web_Material_uhaf032 [file web_material_uhaf032.zip › Figure S3.tif]

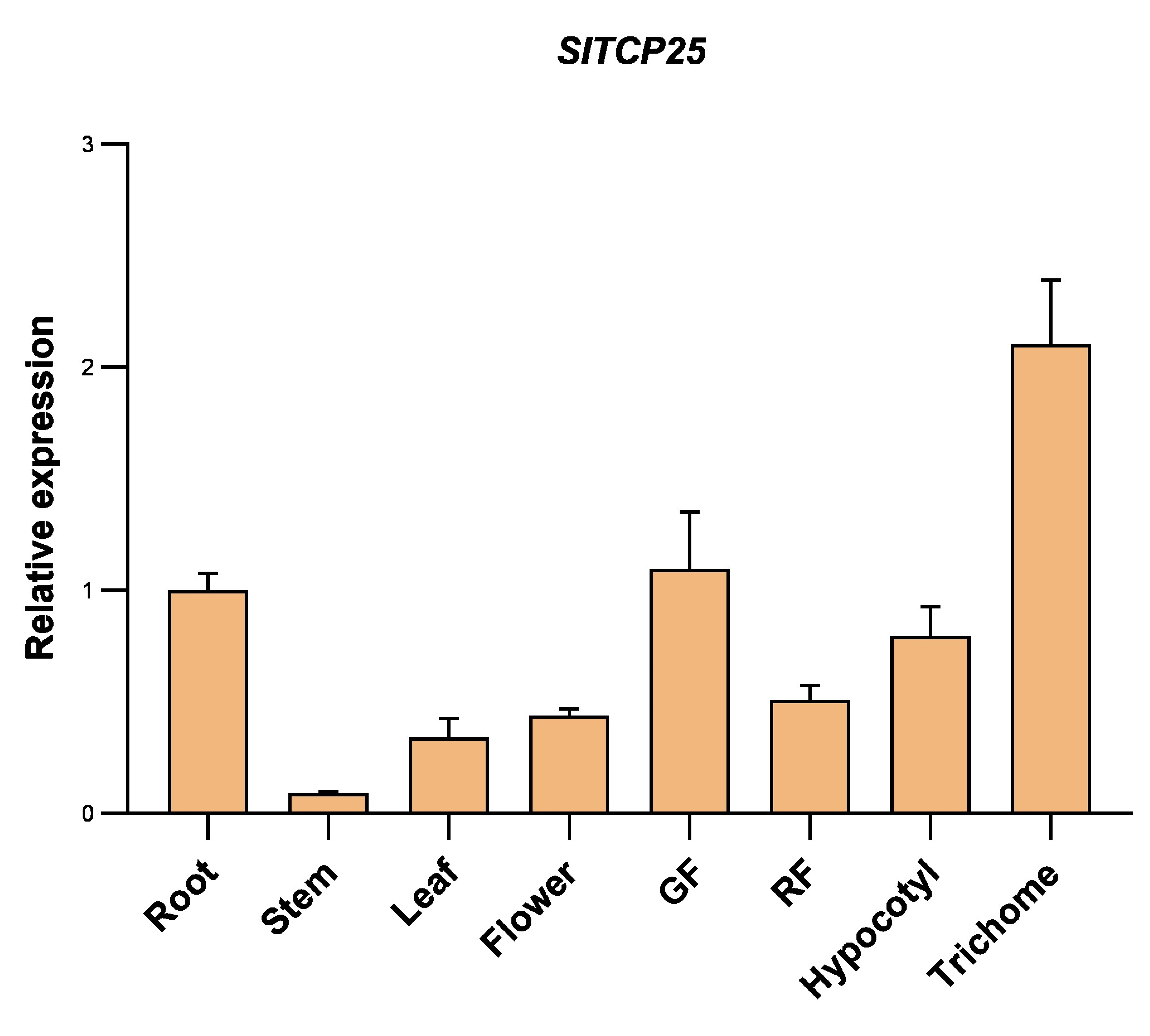

Supplement: Web_Material_uhaf032 [file web_material_uhaf032.zip › Figure S4.tif]

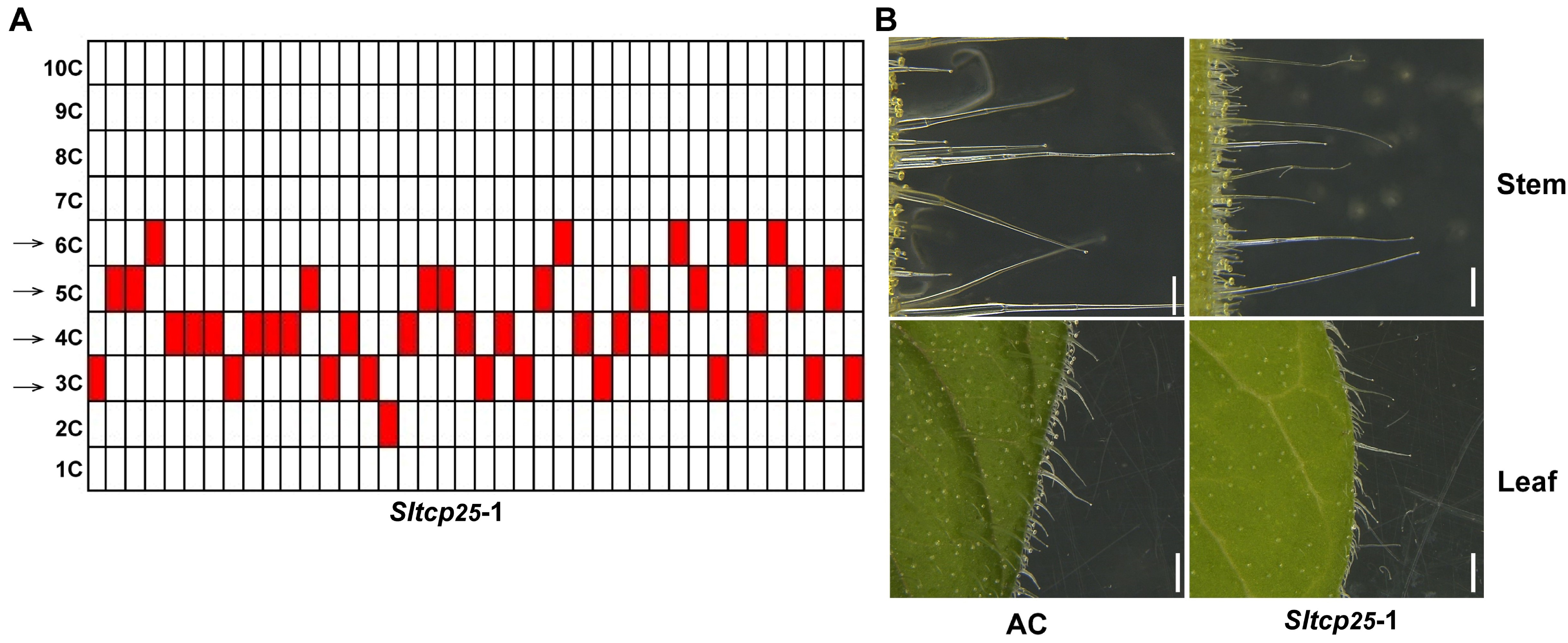

Supplement: Web_Material_uhaf032 [file web_material_uhaf032.zip › Figure S5.tif]

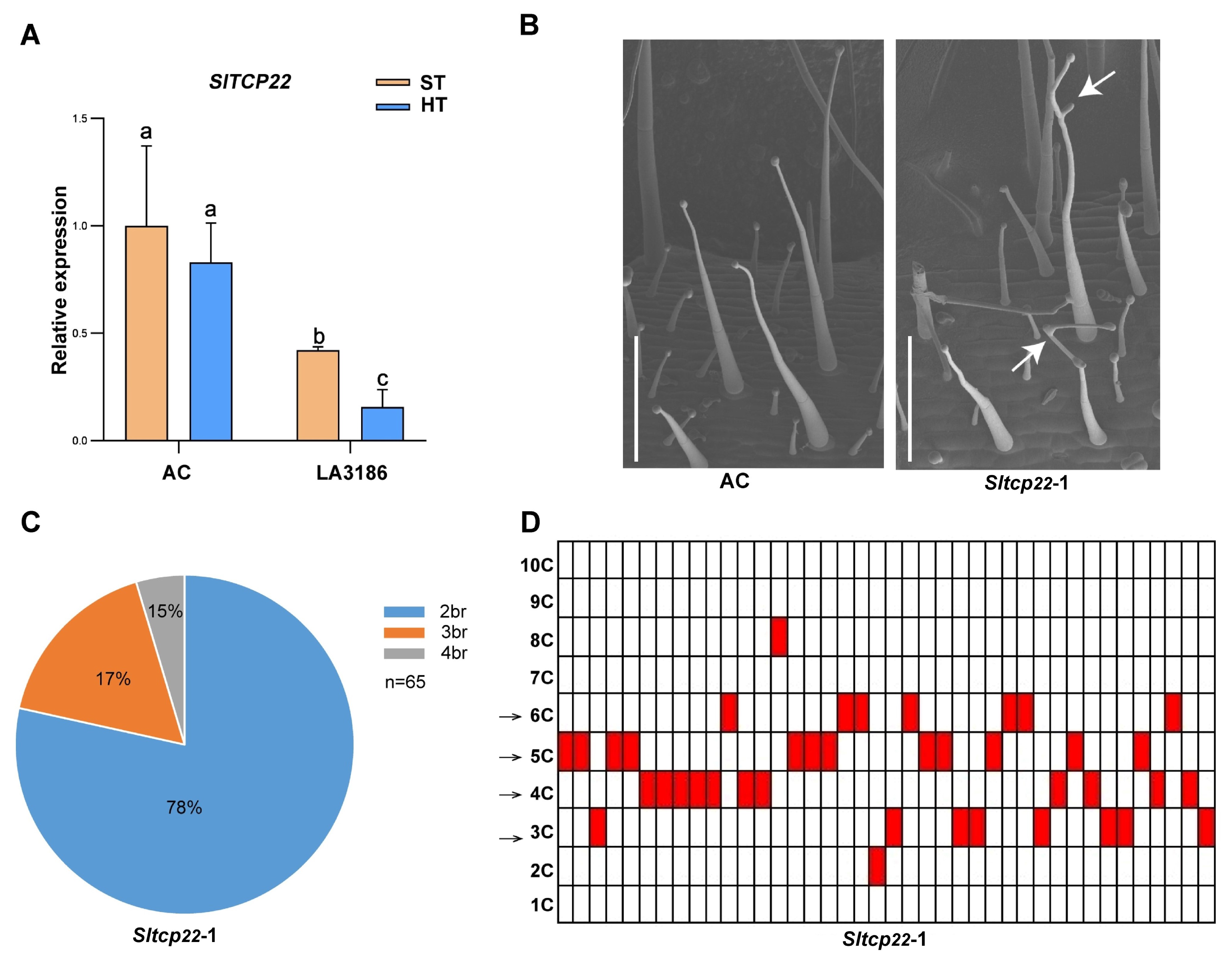

Supplement: Web_Material_uhaf032 [file web_material_uhaf032.zip › Figure S6.tif]

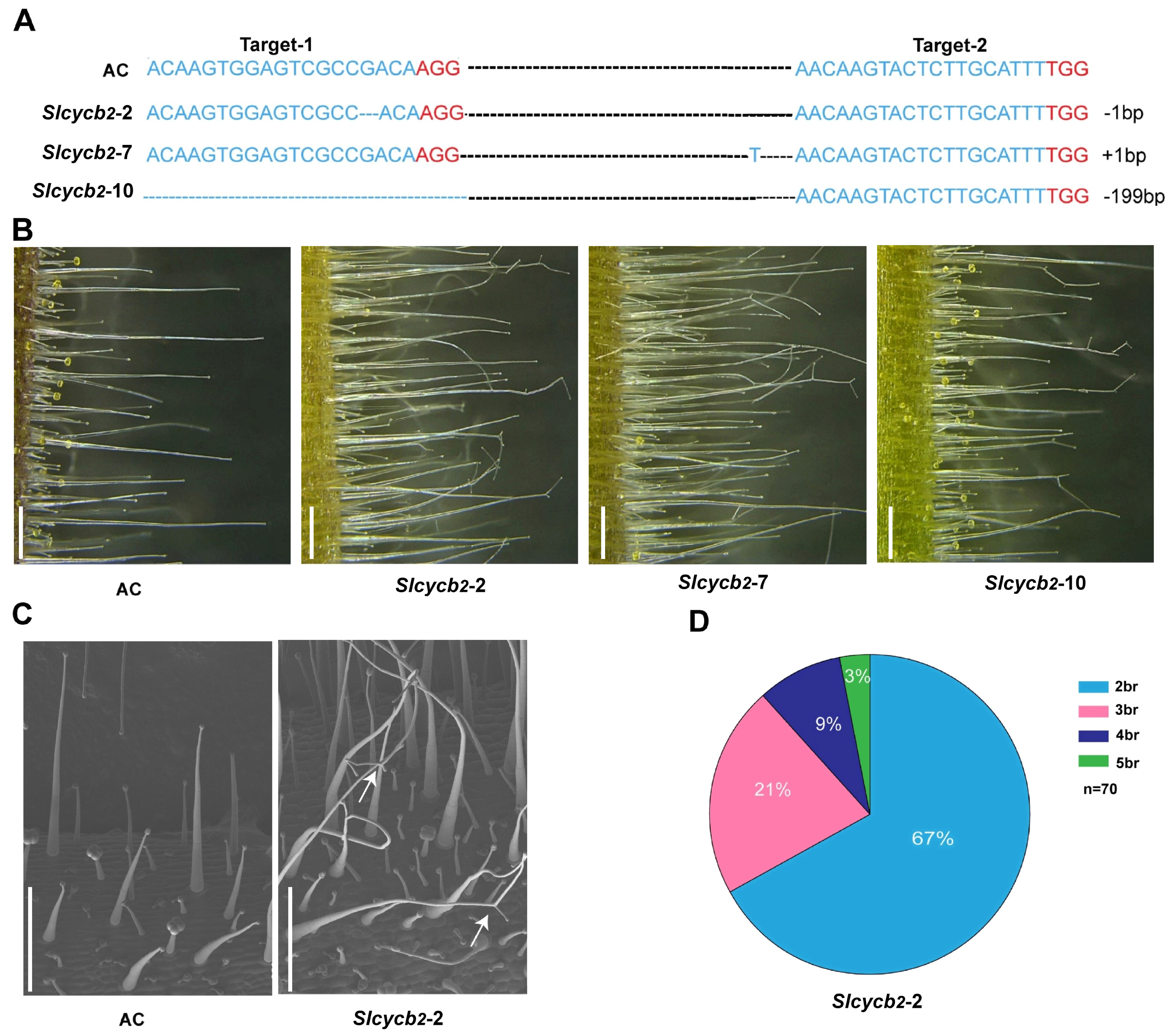

Supplement: Web_Material_uhaf032 [file web_material_uhaf032.zip › Figure S7.tif]

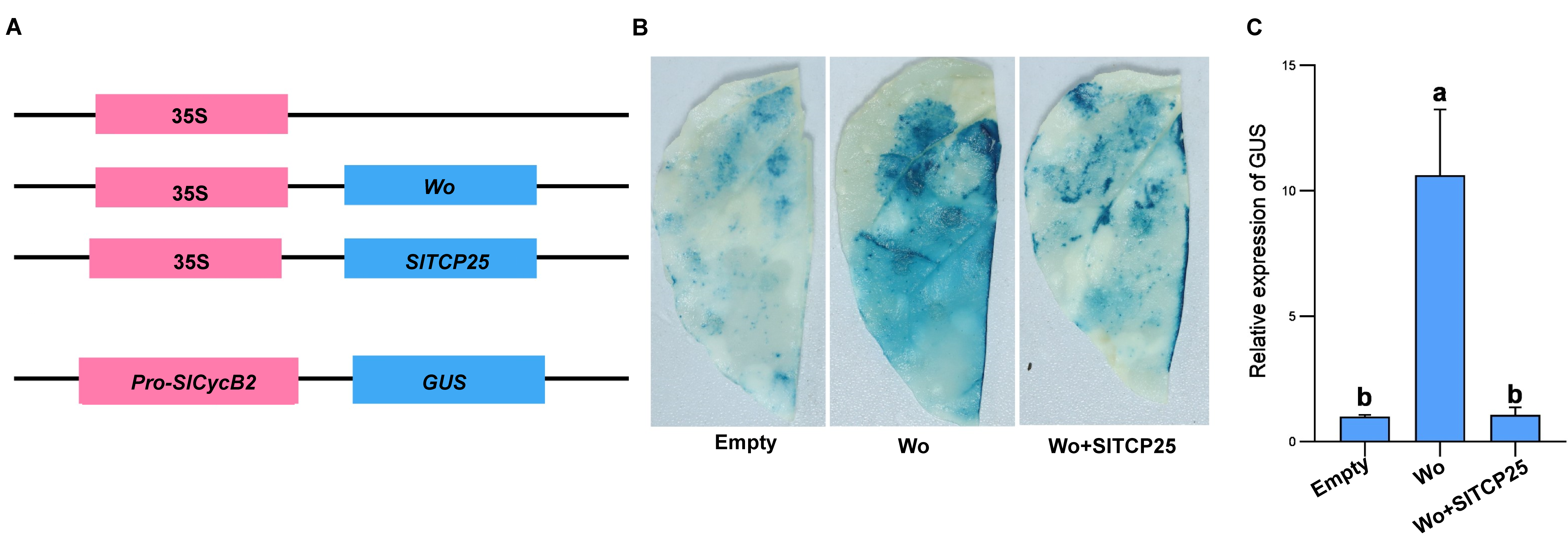

Supplement: Web_Material_uhaf032 [file web_material_uhaf032.zip › Figure S8.tif]
